# Targeting cancer glycosylation repolarizes tumor-associated macrophages allowing effective immune checkpoint blockade

**DOI:** 10.1126/scitranslmed.abj1270

**Published:** 2022-11-02

**Authors:** Michal A. Stanczak, Natalia Rodrigues Mantuano, Nicole Kirchhammer, David E. Sanin, Francis Jacob, Ricardo Coelho, Arun V. Everest-Dass, Jinyu Wang, Marcel P. Trefny, Gianni Monaco, Anne Bärenwaldt, Melissa A. Gray, Adam Petrone, Abhishek S. Kashyap, Katharina Glatz, Benjamin Kasenda, Karl Normington, James Broderick, Li Peng, Oliver M.T. Pearce, Erika L. Pearce, Carolyn R. Bertozzi, Alfred Zippelius, Heinz Läubli

**Affiliations:** 1 Department of Biomedicine, University Hospital and University of Basel, 4031 Basel, Switzerland.; 2 Bloomberg-Kimmel Institute for Cancer Immunotherapy at Johns Hopkins, Baltimore, MD 21287, USA.; 3 Max Planck Institute of Immunobiology and Epigenetics, 79108 Freiburg, Germany.; 4 Institute for Glycomics, Griffith University, Gold Coast Campus, Gold Coast QLD4222, Australia.; 5 Department of Chemistry, Stanford ChEM-H, and Howard Hughes Medical Institute, Stanford University, Stanford, CA 94305, USA.; 6 Palleon Pharmaceuticals, Waltham, MA 02451, USA.; 7 Institute of Pathology, University Hospital Basel, 4031 Basel, Switzerland.; 8- Division of Oncology, Department of Theragnostics, University Hospital Basel, 4031 Basel, Switzerland.; 9 Centre for Tumour Microenvironment, Barts Cancer Institute, Queen Mary University, London EC1M 6BQ, UK.

## Abstract

Immune checkpoint blockade (ICB) has substantially improved the prognosis of patients with cancer, but the majority experiences limited benefit, supporting the need for new therapeutic approaches. Up-regulation of sialic acid–containing glycans, termed hypersialylation, is a common feature of cancer-associated glycosylation, driving disease progression and immune escape through the engagement of Siglec receptors on tumor-infiltrating immune cells. Here, we show that tumor sialylation correlates with distinct immune states and reduced survival in human cancers. The targeted removal of Siglec ligands in the tumor microenvironment, using an antibody-sialidase conjugate, enhanced antitumor immunity and halted tumor progression in several murine models. Using single-cell RNA sequencing, we revealed that desialylation repolarized tumor-associated macrophages (TAMs). We also identified Siglec-E as the main receptor for hypersialylation on TAMs. Last, we found that genetic and therapeutic desialylation, as well as loss of Siglec-E, enhanced the efficacy of ICB. Thus, therapeutic desialylation represents an immunotherapeutic approach to reshape macrophage phenotypes and augment the adaptive antitumor immune response.

## INTRODUCTION

Cancer immunotherapy using immune checkpoint blockade (ICB), including antibodies blocking cytotoxic T lymphocyte protein 4 (CTLA-4) and programmed cell death protein 1 [PD-(L)1], has improved the outcomes of patients with cancer, although overall, only a minority benefit from the currently available ICB (1, 2). New target pathways are under investigation, and combination approaches with CTLA-4– and PD-(L)1–blocking agents have shown promising preclinical and early clinical activity (3). The up-regulation of sialic acid–containing glycans in the tumor microenvironment, termed tumor hypersialylation, contributes to the establishment of an immunosuppressive milieu and dampens antitumor immune responses through the engagement of immunomodulatory Siglecs expressed on tumor-infiltrating immune cells (4-7). Recent work has suggested the sialoglycan-Siglec axis as a new immune checkpoint that can be targeted to drive innate and adaptive antitumor immunity (8). However, given the existence of multiple Siglecs and their broad pattern of expression in the immune system, the exact mechanism remains unclear.

The expression of inhibitory CD33-related Siglecs, including human Siglec-7 and Siglec-9 and murine Siglec-E, on tumor-associated macrophages (TAMs) was shown to support cancer progression by driving macrophage polarization toward the tumor-promoting M2 phenotype (9-13). Similarly, natural killer (NK) cell–mediated killing of tumor cells can be blocked in a dose-dependent manner by the interactions between tumor sialoglycans and Siglec-7 and Siglec-9 on human NK cells (14, 15). Recent work by us and others identified Siglec-9 as an inhibitory receptor expressed on tumor-infiltrating T cells in different cancers, including non–small cell lung cancer, epithelial ovarian cancer, colorectal cancer, and melanoma (5, 16-18). We previously showed that genetic desialylation of tumor cells halts tumor growth and leads to improved antitumor immunity in mouse models (5) and demonstrated that treatment with sialidase can achieve similar results (19). Although a growing body of evidence supports the potential of targeting tumor sialylation, the feasibility of therapeutic interventions and enhancement of classical ICB remained to be demonstrated.

Here, we investigated the cellular and molecular mechanisms by which therapeutic desialylation augments antitumor immunity and halts tumor growth. We identified Siglec-E expression on TAMs as a mediator of the effects of desialylation. Last, we demonstrated improved efficacy of therapeutic desialylation in combination with ICB.

## RESULTS

### Tumor sialylation is associated with immune suppression and reduced survival in patients with cancer

**Fig. 1.**
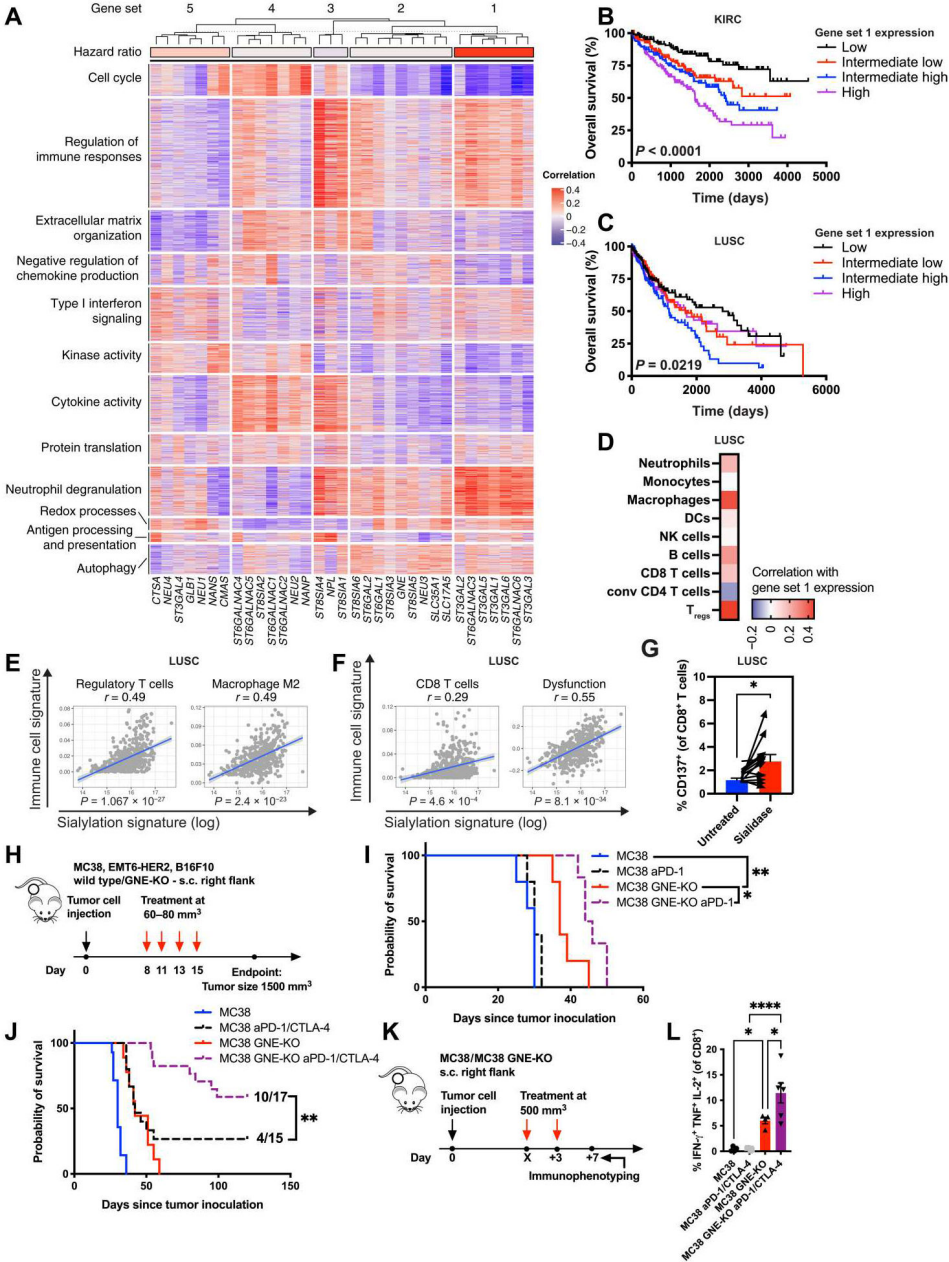
Tumor sialylation is associated with immune suppression and reduced survival in patients with cancer. **(A)** Clustering of correlations between the expression of sialic acid–modifying enzymes and the expression of immune genes in all solid cancers from The Cancer Genome Atlas (TCGA) database. **(B)** Kaplan-Meier survival curve of patients with clear cell renal carcinoma (KIRC) or **(C)** squamous cell carcinoma of the lung (LUSC) is shown, divided into quartiles on the basis of their low, intermediate-low, intermediate-high, and high expression of gene set 1, using TCGA data. **(D)** Correlations between gene set 1 expression and gene signatures of tumor-infiltrating immune cell types are shown for all patients with LUSC from TCGA data. *r* values are shown on a color scale, from blue to red. **(E)** Dot plots displaying correlations between gene set 1 expression and signatures of T_regs_ and M2 macrophages (*P* < 0.01). **(F)** Dot plots displaying correlations between gene set 1 expression and signatures of CD8^+^ T cells and T cell dysfunction in cancer (*P* < 0.01). **(G)** Expression of CD137 (4-1BB) was measured on CD8^+^ T cells from primary LUSC samples from individual patients after a 48-hour incubation with or without *V. cholerae* sialidase (*n* = 13). **(H)** Experimental design: Mice bearing subcutaneous or intramammary wild-type or GNE-KO tumors were treated intraperitoneally with four doses of anti–PD-1 or anti–CTLA-4 antibodies (10 mg/kg) individually or in combination, beginning at a tumor size of about 80 mm^3^. **(I)** Effect of PD-1 blockade on the survival of mice bearing subcutaneous wild-type or GNE-KO MC38 tumors (*n* = 5 to 6 mice per group). **(J)** Effect of combined PD-1 and CTLA-4 blockade on the survival of mice bearing subcutaneous wild-type or GNE-KO MC38 tumors (*n* = 14 to 17 mice per group). **(K)** Experimental design: Mice bearing established (about 500 mm^3^) subcutaneous wild-type or GNE-KO MC38 tumors were treated intraperitoneally with two doses of anti–PD-1 and anti–CTLA-4 antibodies (10 mg/kg). Seven days after the first treatment, tumors were resected and immunophenotyped. **(L)** Frequency of IFN-γ^+^TNF^+−^IL2^+^ CD8^+^ T cells after restimulation in single-cell suspensions of MC38 wild-type or GNE-KO tumors treated with PD-1 and CTLA-4 blockade (*n* = 4 to 6 mice per group). *n* indicates the number of biological replicates. Error bars represent means ± SEM. Statistical analyses were performed using the log-rank (Mantel-Cox) test for the TCGA survival data or the Gehan-Wilcoxon test for the mouse survival data, followed by Bonferroni’s correction for multiple comparisons. A paired two-tailed Student’s *t* test was used in (G) and a one-way ANOVA followed by a post hoc Šidák correction for multiple comparisons in (L). *P ≤ 0.05, **P ≤ 0.01, and ****P ≤ 0.0001.

As increased tumor sialylation has previously been linked to immune suppression (20, 21), we wanted to test whether the expression of sialic acid–modifying enzymes is associated with distinct immune states in human cancer. To that end, we assembled a set of 34 genes encoding for proteins involved in sialoglycan biosynthesis and adopted a previously published set of 3021 immune genes (22). The expression of sialoglycan genes was correlated with that of the immune genes using the data of all solid cancers from The Cancer Genome Atlas (TCGA) database. *K*-mean clustering was applied to the correlation matrix, and the sialoglycan genes were best clustered into five gene sets. These gene sets were tested for their association with patient survival (Fig. 1A and fig. S1A), leading to the identification of gene set 1, consisting of α-2,3- and α-2,6-sialyltransferases, which was positively correlated with regulation of immune function and reduced cytokine activity. Expression of gene set 1 strongly correlated with reduced survival of patients with several cancer types (fig. S1B), particularly patients with clear cell kidney cancer (KIRC; Fig. 1B) and squamous cell lung cancer (LUSC; Fig. 1C and fig. S1C). To corroborate this finding, we stained a tissue microarray of 75 primary human KIRC samples and corresponding healthy control tissues with a hexameric human Siglec-9 Fc protein (HYDRA, Palleon Pharmaceuticals) (23) to quantify the expression of sialic acid–containing Siglec ligands. Analysis of the linked survival data revealed a worse overall survival in patients with KIRC with increasing Siglec-9 Fc binding (fig. S1, D and E), validating the use of gene set 1 expression as a proxy for hypersialylation and supporting the association with reduced survival.

Then, we correlated the expression of gene set 1 with gene expression signatures of different tumor-infiltrating immune cell types in all patients with LUSC and found the strongest positive correlations with immunosuppressive and tumor-promoting cell types (Fig. 1D), such as regulatory T cells (T_regs_) and TAMs (Fig. 1E). In contrast, the correlation with conventional CD4^+^ T cells was significant (*P* = 0.004) but negative (fig. S1F), and no correlations were found with other myeloid cell types such as dendritic cells (DCs) or monocytes (fig. S1G). Although a small positive correlation could be observed with CD8^+^ T cells, it was weaker than the correlation with an established signature of T cell dysfunction in cancer (Fig. 1F) (24). To test the correlation between tumor sialylation and T cell dysfunction, we cultured primary tumor suspensions from patients with LUSC with or without sialidase. In line with our findings, sialidase treatment led to a significant (*P* = 0.025) increase in CD8 T cell activation, measured by the expression of the activation marker CD137 (Fig. 1G). These data link tumor sialylation to specific changes in immune infiltration, predominantly by immunosuppressive and tumor-promoting cell types and reduced survival of patients.

To corroborate the association between tumor sialylation and T cell dysfunction, we used a mouse tumor model of genetic desialylation in combination with anti–PD-1 and anti–CTLA-4 ICB. We previously showed that the growth of tumors lacking the rate-limiting enzyme for sialic acid biosynthesis, uridine diphosphate–*N*-acetylglucosamine 2-epimerase (GNE), was delayed, and survival of mice prolonged (5). We confirmed the delayed tumor growth of MC38 GNE-knockout (KO) tumors and were able to show the delay to be dependent on CD8^+^ T cells, evidenced by its abrogation when CD8^+^-depleting antibodies were applied (fig. S1, H and I). To test the effect of tumor sialylation on the ability of ICB to reinvigorate tumor-infiltrating CD8 T cells, we subcutaneously injected mice with wild-type or GNE-KO MC38 tumor cells and treated mice bearing palpable tumors with four doses of PD-1–blocking antibodies alone or in combination with CTLA-4–blocking antibodies (Fig. 1H). Treatment of GNE-KO tumors with anti–PD-1 resulted in a stronger reduction in tumor growth and prolonged survival of mice compared to treatment of wild-type tumors (Fig. 1I and fig. S1J). Application of both PD-1 and CTLA-4 blockade led to the rejection of wild-type tumors in 4 of 15 mice (27%), whereas the rejection rate was increased to 10 of 17 (59%) in mice bearing GNE-KO tumors (Fig. 1J and fig. S1K).

To exclude a cell line or mouse strain–specific effect, we used the EMT6-HER2 (human epidermal growth factor receptor-2) mammary carcinoma model injected orthotopically into the mammary fat pads of female BALB/c mice. Again, we observed a delayed tumor growth of GNE-KO tumors compared to wild-type tumors and an increased rejection rate of GNE-KO tumors treated with anti–PD-1 antibodies compared to wild-type tumors (50% versus 33%; fig. S1, L and M). In addition, we applied the poorly immunogenic and highly aggressive B16F10 melanoma model, again demonstrating that GNE-KO delayed tumor growth, prolonged survival, and increased sensitivity to PD-1/CTLA-4 blockade, resulting in a more pronounced delay in tumor growth and a longer survival of mice bearing GNE-KO tumors (fig. S1, N and O). To functionally test the reinvigoration of CD8 T cell function by desialylation in combination with ICB, we treated mice bearing established (about 500 mm^3^) wild-type or GNE-KO MC38 tumors with two doses of PD-1– and CTLA-4–blocking antibodies and isolated tumor-infiltrating immune cells 7 days after the first treatment (Fig. 1K and fig. S1, P and Q). T cells from the single-cell suspensions of those tumors were restimulated in vitro and intracellularly stained for the expression of CD8^+^ effector T cell cytokines. Although ICB alone increased the frequencies of both interferon-γ–positive (IFN-γ^+^) and IFN-γ^+^ tumor necrosis factor–positive (TNF^+^) CD8^+^ T cells (fig. S1R), desialylation augmented T cell activation, particularly when assessing the frequency of multifunctional IFN-γ^+^TNF^+^ interleukin-2–positive (IL-2^+^) CD8^+^ T cells, which were not induced by ICB alone (Fig. 1L). These findings support the association between tumor sialylation, immunosuppression, and dysfunction of CD8 T cells and also demonstrate an increased reinvigoration of tumor-infiltrating T cells by ICB in desialylated tumors.

### Tumor-targeted sialidase effectively desialylates the tumor microenvironment

**Fig. 2.**
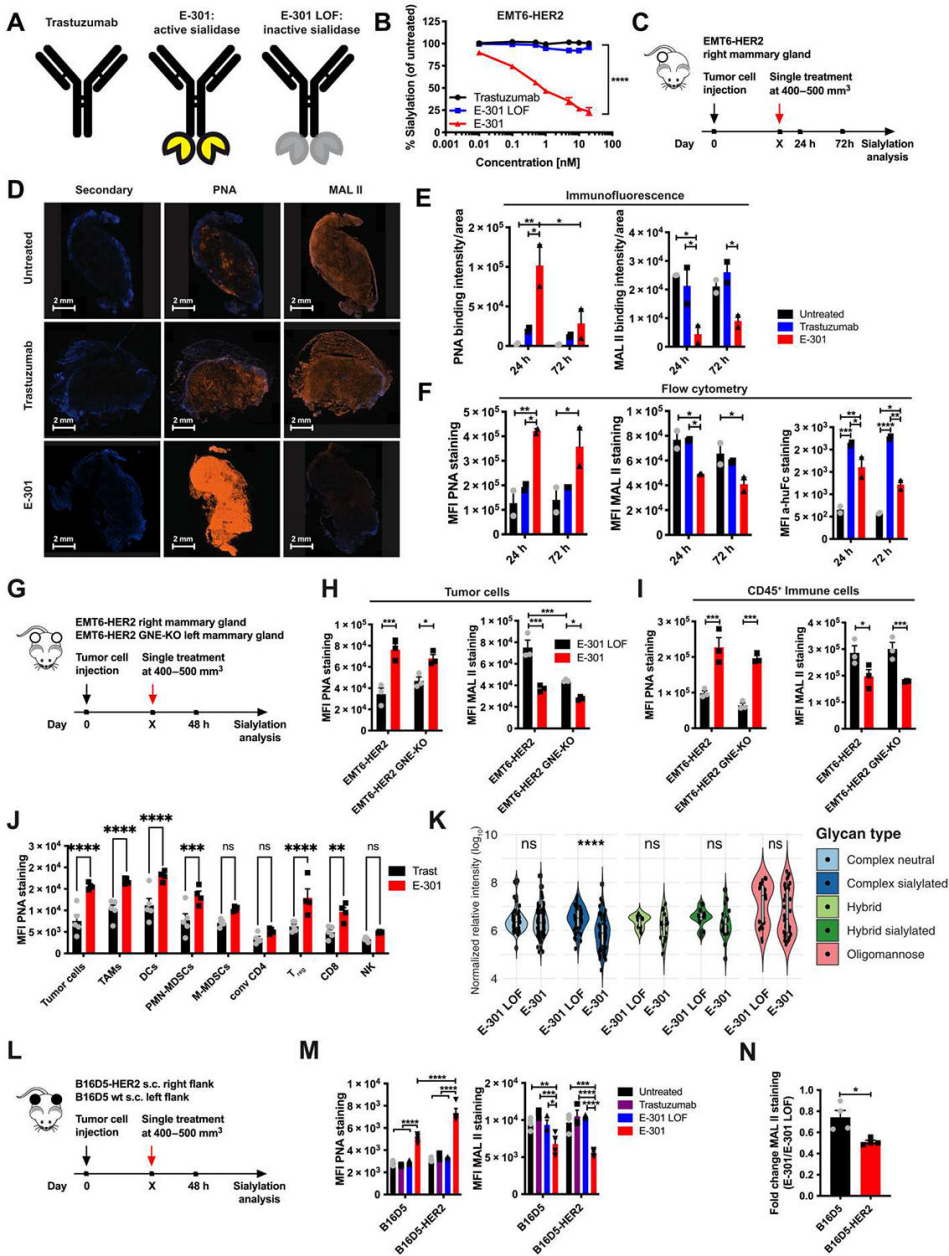
Tumor-targeted sialidase effectively desialylates the tumor microenvironment. **(A)** Schematic representation of the tumor-targeted sialidase constructs: trastuzumab, the trastuzumab-sialidase conjugate E-301, and the loss-of-function (LOF) mutated, enzymatically inactive version E-301 LOF. **(B)** In vitro titration of trastuzumab, E-301, and E-301 LOF on EMT6-HER2 cells. Desialylation was assessed by PNA staining 24 hours after treatment relative to that after maximal desialylation (*n* = 3). **(C)** Experimental setup to test in vivo desialylation: Mice bearing established (500-mm^3^) intramammary EMT6-HER2 tumors were treated with a single dose of PBS, trastuzumab, or E-301 [10 mg/kg, intraperitoneally (i.p.)], and desialylation was assessed at 24 and 72 hours after treatment. **(D)** Representative immunofluorescence images of untreated, trastuzumab-treated, and E-301–treated EMT6-HER2 tumors at 24 hours after treatment, stained with anti-human Fc secondary, PNA, and MAL II. Scale bars, 2 mm. **(E)** Quantification of immunofluorescence staining with PNA and MAL II. The sum of the staining intensity was normalized to the respective 4′,6-diamindino-2-phenylindole–stained area. **(F)** Flow cytometric analysis of intramammary EMT6-HER2 tumor sialylation [same tumors as in (E)] by lectin staining of tumor cell suspensions at 24 and 72 hours after treatment. Geometric mean fluorescence intensities (MFIs) of PNA, MAL II, and secondary anti-human Fc staining are shown (D to F) (*n* = 2 mice per group). **(G)** Experimental setup comparing desialylation of intramammary wild-type and GNE-KO EMT6-HER2 tumors: Mice bearing established (500-mm^3^) intramammary wild-type or GNE-KO EMT6-HER2 tumors were treated with a single dose of E-301 LOF or E-301 (10 mg/kg, i.p.), and desialylation was assessed at 48 hours after treatment. **(H)** Geometric MFIs of PNA and MAL II staining of tumor cells. (**I**) Geometric MFIs of PNA and MAL II staining of tumor-infiltrating CD45^+^ cells (H and I) (*n* = 3 mice per group). **(J)** Desialylation of different intratumoral immune cell populations from EMT6-HER2 tumors by E-301 was measured by PNA staining 48 hours after treatment with trastuzumab or E-301 (*n* = 4 to 5 mice per group). Conv, conventional. **(K)** Liquid chromatography–mass spectrometry–based N-glycan analysis of EMT6-HER2 cells after overnight in vitro treatment with E-301 LOF or E-301 (*n* = 3 samples per group). **(L)** Experimental setup comparing desialylation of subcutaneous B16D5 and B16D5-HER2 tumors at 48 hours after intraperitoneal treatment with a single dose of trastuzumab, E-301, or E-301 LOF (10 mg/kg). **(M)** Geometric MFIs of PNA and MAL II staining of tumor cells. **(N)** Fold change in the geometric MFI of MAL II staining after E-301 treatment relative to E-301 LOF treatment (M and N) (*n* = 4 mice per group). *N* indicates the number of biological replicates. Error bars represent means ± SEM. Statistical analyses were performed using two-way ANOVAs followed by post hoc Šidák corrections for multiple comparisons, and an unpaired two-tailed Student’s *t* test was used to assess fold change differences in (N). ns, not significant; *P ≤ 0.05, **P ≤ 0.01, ***P ≤ 0.001, and ****P ≤ 0.0001.

Next, we aimed to study the cellular and molecular mechanisms underlying sialic acid–mediated immune suppression in greater detail using therapeutic desialylation in different mouse tumor models. To achieve tumor-specific desialylation in vivo, we used an anti-body-sialidase fusion protein to target the enzyme to the tumor microenvironment, similarly to a previously used sialidase-antibody construct (19). Specifically, we used the antibody trastuzumab, which recognizes HER2. The trastuzumab-sialidase construct, termed E-301, was bioengineered on the Enzyme-Antibody-Glycan-Ligand-Editing platform (Palleon Pharmaceuticals) (25) by fusing two sialidase domains to the C-terminal Fc region of trastuzumab. As controls, we used unmodified trastuzumab, as well as an enzymatically inactive E-301 variant carrying two loss-of-function (LOF) mutations in the catalytic sites of its sialidases (E-301 LOF; Fig. 2A). We confirmed dose-dependent desialylation of HER2-expressing EMT6 mammary carcinoma cells (EMT6-HER2) by E-301 but not by trastuzumab or E-301 LOF (Fig. 2B and fig. S2A). To evaluate tumor cell desialylation in vivo, we established orthotopic EMT6-HER2 tumors in BALB/c mice until they reached a size of 400 to 500 mm^3^, upon which the mice were treated systemically with a single intraperitoneally administered dose of 10 mg/kg of E-301 or trastuzumab (Fig. 2C). We observed increased staining with peanut agglutinin (PNA), detecting galactosyl residues uncovered by the removal of sialic acids, and decreased staining with *Maackia amurensis* lectin II (MAL II), binding to α-2,3-sialic acids, in the E-301–treated tumors compared with the trastuzumab-treated and untreated tumors. Desialylation was confirmed both by immunofluorescence (Fig. 2, D and E) and by flow cytometry (Fig. 2F) and was most pronounced at 24 hours but still detectable at 72 hours after injection (fig. S2, B and C). Staining with a Siglec-E Fc fusion protein at 72 hours after treatment confirmed the loss of Siglec ligands after E-301 treatment (fig. S2D). Flow cytometric staining for human Fc not only further confirmed successful targeting of E-301 into the tumor but also suggested an accelerated clearance of E-301 compared to trastuzumab (Fig. 2F and fig. S2E). In addition, staining with *Sambucus nigra* lectin (SNA) showed a clear reduction of α-2,6–linked sialic acids in tumors treated with E-301 (fig. S2F). Our results confirm that targeting of sialidase to tumor cells results in a strong but transient reduction of sialoglycan abundance in vivo.

Then, we wanted to assess whether direct interactions between the sialidase′s glycan-binding domains and tumor sialoglycans might influence sialidase targeting. To this end, we used EMT6-HER2 cells lacking GNE (GNE-KO) and confirmed comparable expression of the HER2 antigen (fig. S2G). We then injected wild-type EMT6-HER2 and EMT6-HER2 GNE-KO cells into opposing mammary glands of individual mice and again treated mice bearing established tumors with a single intraperitoneal dose of E-301 or E-301 LOF (Fig. 2G). At 48 hours after E-301 treatment, the wild-type EMT6-HER2 tumors showed strong desialylation, as evidenced by an increase in PNA and a decrease in MAL II staining; the GNE-KO tumors, although already presenting reduced sialylation at baseline, showed a slight further desialylation after treatment (Fig. 2H and fig. S2H), possibly reflecting scavenging of sialic acids form the environment. In contrast, CD45^+^ immune cells showed an equally strong loss of sialylation in both wild-type and GNE-KO tumors (Fig. 2I). A similar degree of staining with the mannosebinding lectin Concavalin A (ConA) across all groups confirmed the observed changes in cancer glycosylation to be specific to sialic acid–containing glycans (fig. S2I).

To further delineate which immune cells are desialylated by sialidase treatment, we combined PNA staining with a comprehensive flow cytometric immune phenotyping of EMT6-HER2 tumors 48 hours after E-301 treatment. We found desialylation of nearly all immune cells, including TAMs, DCs, polymorphonuclear myeloid-derived suppressor cells (PMN-MDSCs), T_regs_, and CD8^+^ T cells. In contrast, monocyte (M)–MDSCs, conventional CD4^+^ T cells, and NK cells did not exhibit increased PNA staining (Fig. 2J).

Next, we performed liquid chromatography–mass spectrometry (MS)–based N-glycan analysis on in vitro E-301–treated EMT6-HER2 tumor cells to characterize the effect of sialidase treatment in further detail. A reduction in complex sialylated N-glycans was observed in E-301–treated EMT6-HER2 cells compared to E-301 LOF–treated controls (Fig. 2K), as well as in GNE-KO B16F10 cells compared to wild-type controls, whereas a reduction in both complex and hybrid sialylated N-glycans was observed in GNE-KO EMT6-HER2 and MC38 cells compared to their respective wild-type controls (fig. S2, J to L).

We further assessed the capacity of E-301 to target and desialylate HER2-expressing melanoma B16D5 (B16D5-HER2) tumors. C57BL/6 mice were subcutaneously injected with B16D5-HER2 tumor cells in the right and parental B16D5 cells in the left flank (Fig. 2L). Although desialylation was detected in both tumor types when treated with E-301, it was more pronounced in the B16D5-HER2 tumors compared to the B16D5 tumors (Fig. 2, M and N, and fig. S2M). Whereas the presence of all compounds could be detected in the B16D5-HER2 tumors by staining for human Fc, no staining was observed in the B16D5 tumors (fig. S2N), suggesting a prolonged retention of the constructs in the HER2-expressing tumors. Again, staining with ConA confirmed the sialic acid specificity of these changes (fig. S2O). These findings demonstrate the feasibility of using E-301 to target systemically administered sialidase into HER2-expressing tumors and confirm effective desialylation of the tumor microenvironment.

### Tumor-targeted sialidase inhibits tumor growth by activating the adaptive immune system

**Fig. 3.**
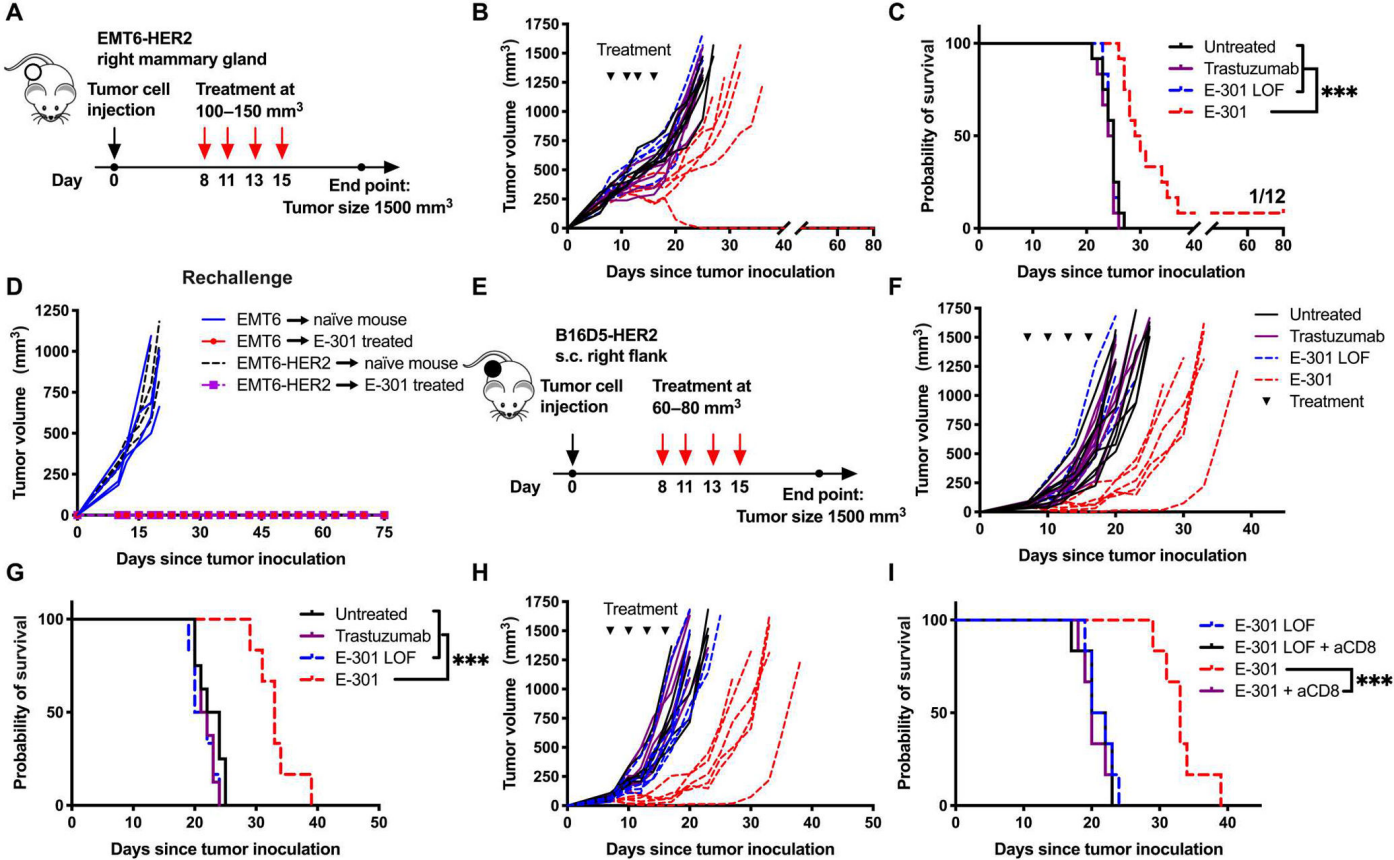
Tumor-targeted sialidase inhibits tumor growth by activating the adaptive immune system. **(A)** Experimental setup: Mice bearing intramammary EMT6-HER2 tumors were treated intraperitoneally with four doses of trastuzumab, E-301 LOF, or E-301 (10 mg/kg), beginning at a tumor size of about 100 mm^3^. **(B)** Growth of individual intramammary EMT6-HER2 tumors treated with trastuzumab, E-301 LOF, or E-301 (*n* = 6 to 8 mice per group). **(C)** Survival of mice bearing intramammary EMT6-HER2 tumors treated with trastuzumab, E-301 LOF, or E-301 (pooled from two experiments, *n* = 12 mice per group). **(D)** Rechallenge of the tumor-free mouse from (C) and tumor-naïve control mice with subcutaneous EMT6 or EMT6-HER2 tumor cells in each flank, respectively (*n* = 1 to 4 mice per group). **(E)** Experimental setup: Mice bearing subcutaneous B16D5-HER2 tumors were treated with four doses of trastuzumab, E-301 LOF, or E-301 (10 mg/kg, i.p.), with the first dose administered once the tumor size reached about 80 mm^3^. **(F)** Growth of individual subcutaneous B16D5-HER2 tumors treated with trastuzumab, E-301 LOF, or E-301. **(G)** Survival of mice bearing B16D5-HER2 tumors treated with trastuzumab, E-301 LOF, or E-301 (F and G) (*n* = 6 to 8 mice per group). **(H)** Growth of individual B16D5-HER2 tumors treated with E-301 LOF or E-301 after CD8^+^ T cell depletion. **(I)** Impact of CD8^+^ T cell depletion on the survival of mice bearing B16D5-HER2 tumors treated with E-301 LOF or E-301 (H and I) (*n* = 6 to 8 mice per group). *n* indicates the number of biological replicates. Statistical analyses were performed using the log-rank (Mantel-Cox) test or the Gehan-Wilcoxon test, followed by Bonferroni’s correction for multiple comparisons for all survival analyses. ***P ≤ 0.001.

Our next aim was to test the therapeutic efficacy of systemic E-301 treatment in different HER2-expressing tumor models. As a first model, we used the orthotopic, intramammary EMT6-HER2 model (Fig. 3A). Tumor growth was delayed and survival prolonged in mice treated with four doses of E-301, and 1 tumor of 12 was rejected (Fig. 3, B and C). Although only one mouse showed complete tumor rejection, rechallenge with both wild-type EMT6 and EMT6-HER2 cells, in opposing flanks, resulted in no tumor growth on either side, indicating the development of immunological memory not restricted to the antigenicity of HER2 (Fig. 3D). The body weights of treated mice increased similarly over the course of the experiment among all treatment groups, suggesting no signs of acute toxicity (fig. S3A). Similarly, B16D5-HER2 tumor growth was clearly delayed and survival prolonged after E-301 monotherapy (Fig. 3, E to G). Again, no signs of acute toxicity of the treatment could be observed (fig. S3B). Seeing the development of immunological memory after E-301–induced tumor rejection, we performed an antibody-mediated depletion experiment to test the involvement of the adaptive immune system. We found that depletion of CD8^+^ T cells fully abrogated the delay in tumor growth by E-301 (Fig. 3, H and I). These data show that therapeutic tumor-targeted desialylation is efficacious in delaying tumor growth in different mouse models by activating the adaptive immune system and induces the generation of immunological memory.

### Therapeutic desialylation repolarizes TAMs

**Fig. 4.**
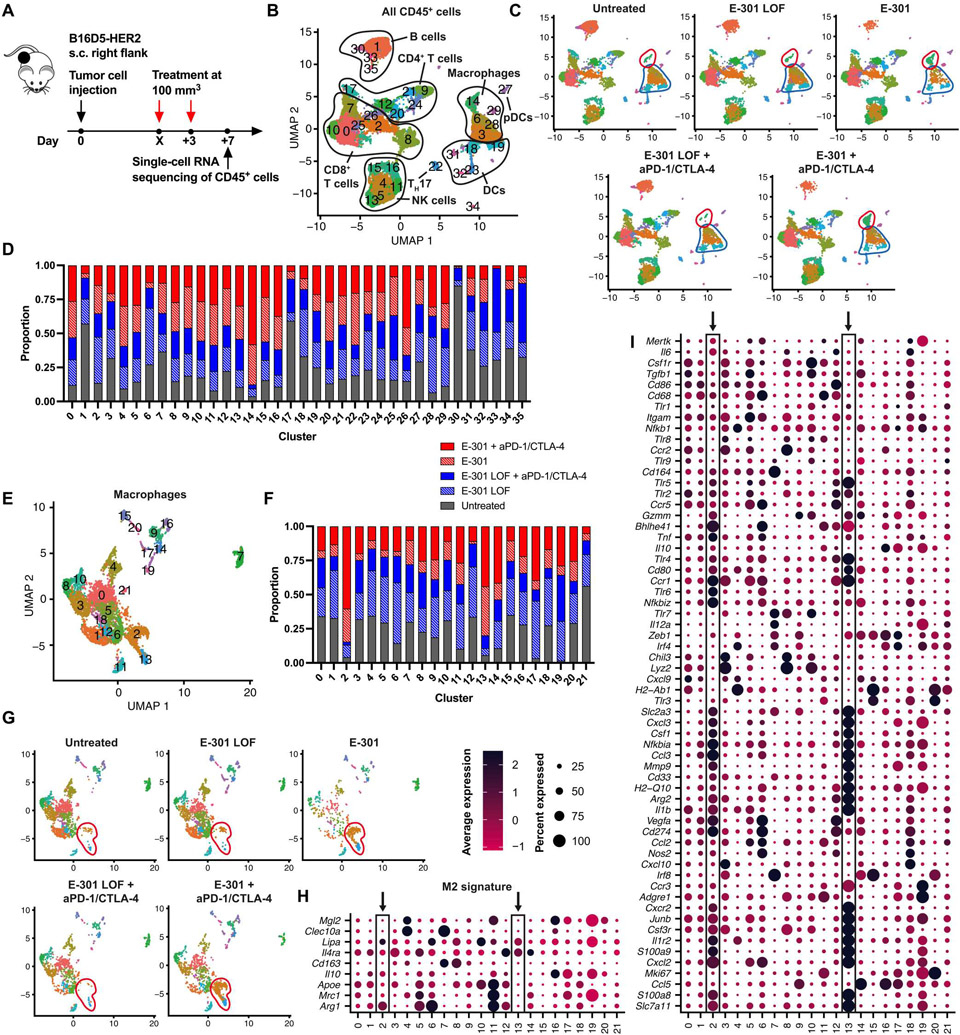
Therapeutic desialylation repolarizes TAMs. **(A)** Experimental setup for single-cell RNA sequencing (scRNA-seq) of immune infiltrates after E-301 treatment: Mice bearing palpable (100-mm^3^) subcutaneous B16D5-HER2 tumors were treated with two doses of E-301 LOF or E-301 (10 mg/kg), alone or in combination with anti–PD-1/CTLA-4 antibodies. CD45^+^ tumor-infiltrating immune cells were isolated and sorted 7 days after the first injection for scRNA-seq. **(B)** scRNA-seq gene expression data were processed, sorted into clusters, and are presented in a dimensional reduction projection (UMAP), showing the different identified immune cell populations. Labels have been added on the basis of the expression of marker genes. **(C)** UMAP projections are shown separated by condition. Clusters 3 and 6 are highlighted in blue, and cluster 14 is highlighted in red. **(D)** Contribution of each condition to each cluster of CD45^+^ cells. **(E)** Subclustering of all macrophages. **(F)** Contribution of each condition to each macrophage cluster. **(G)** UMAP projections of macrophages are shown separated by condition. Clusters 2 and 13 are highlighted in red. (**H** and **I**) Dot plot representation of differentially expressed genes between the macrophage clusters showing genes characteristic for M2 polarization (H) or reflecting more general macrophage function (I). Size reflects the percentage of each cluster expressing a given gene, and average-scaled expression is indicated on the color gradient. Clusters 2 and 13 are boxed in and highlighted with arrows (A to I) (*n* = 5 pooled mice per condition).

To dissect the cellular and molecular mechanism by which targeted desialylation augments anticancer immune responses, we injected mice bearing palpable subcutaneous B16D5-HER2 tumors intraperitoneally with two doses of either E-301 or E-301 LOF alone or in combination with PD-1– and CTLA-4–blocking antibodies and performed single-cell RNA sequencing (scRNA-seq) on CD45^+^ tumor-infiltrating immune cells 7 days after the first treatment (Fig. 4A). Globally, treatment with E-301 induced distinct changes in populations of both myeloid cells and lymphocytes (Fig. 4, B to D). Most prominently, E-301 affected macrophage populations, resulting in decreased frequencies of clusters 3 and 6 (Fig. 4C, highlighted in blue) and an increase in cluster 14 (Fig. 4C, highlighted in red). Subsetting and reclustering of all macrophages yielded 21 TAM clusters (Fig. 4E), which again showed notable differences after desialylation (Fig. 4, F and G). Pathway enrichment analysis among all macrophages showed increases in pathways related to cell migration, as well as the response to chemokines and proinflammatory cytokines in E-301–treated tumors (fig. S4A). Our differential gene expression analysis revealed that tumors from untreated and E-301 LOF–treated mice predominantly contained immunosuppressive TAMs expressing a high abundance of genes characteristic of alternatively activated (M2-polarized) and proangiogenic macrophages, such as *Arg1* or *Mrc1* (CD206; clusters 5, 6, and 11; Fig. 4H and fig. S4B). In contrast, E-301–treated tumors contained fewer immunosuppressive TAMs and instead displayed a distinct shift toward macrophages predominantly expressing antitumoral effector molecules, such as *Il1b, Tnf, Cd80*, and *Arg2* (Fig. 4I, clusters 2 and 13, highlighted with black boxes).

Treatment with E-301 also affected intratumoral NK and T cell populations (fig. S4, C to G). Among NK cells (fig. S4C), desialylation increased the frequencies of all antitumoral NK cell subsets (fig. S4, D and E). Similarly, analysis of all T cells (fig. S4F) revealed decreases in naïve CD4^+^ T cells (CD4^+^ T_N_, cluster 7) and exhausted CD8^+^ T cells (CD8^+^ T_EX_, cluster 8) and the expansion of effector CD8^+^ T cells (CD8^+^ T_EFF_, clusters 5 and 13) and both T helper 1 (T_H_1) and T_H_2 CD4^+^ T cells (CD4^+^ T_H_1 and T_H_2, clusters 4 and 6; fig. S4, G and H).

### Tumor desialylation repolarizes TAMs in murine and human tumors

**Fig. 5.**
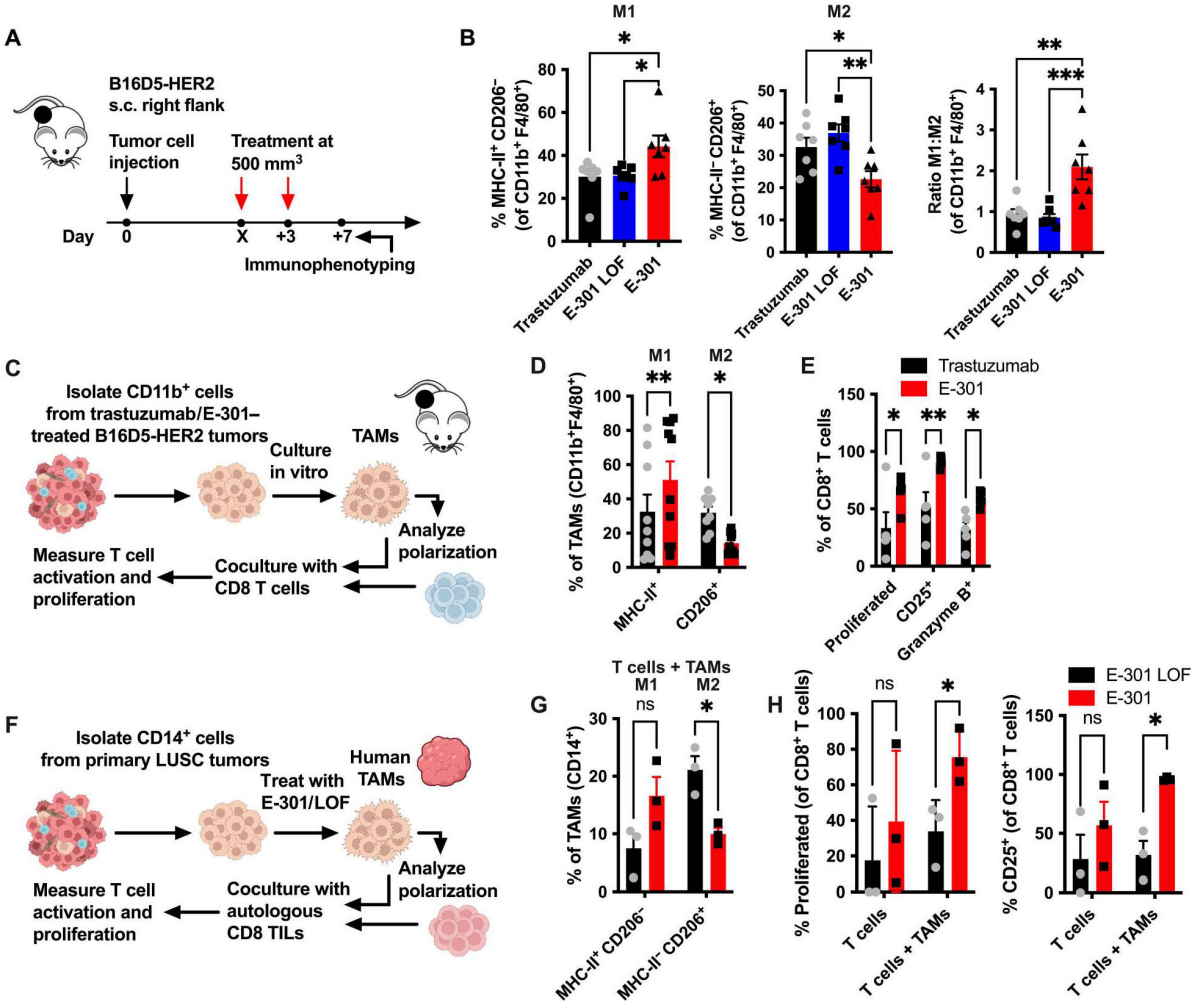
Tumor desialylation repolarizes TAMs in murine and human tumors. **(A)** Experimental setup to phenotype changes in immune infiltrates after E-301 treatment: Mice bearing established (500-mm^3^) subcutaneous B16D5-HER2 tumors were treated with two doses of trastuzumab, E-301 LOF, or E-301 (10 mg/kg, i.p.) and immune infiltrates analyzed after 7 days by flow cytometry. **(B)** Frequencies of MHC-II^+^CD206^−^ (M1) and MHC-II^−^CD206^+^ (M2) cells among CD11b^+^F4/80^+^ tumor-associated macrophages (TAMs) are shown, as well as the ratio of M1 to M2 TAMs (*n* = 7). **(C)** Experimental setup for in vitro coculture of primary mouse CD11b^+^ TAMs and CD8 T cells. Mice carrying established subcutaneous B16D5-HER2 tumors were treated intraperitoneally with two doses of trastuzumab or E-301 (10 mg/kg), and CD11b^+^ TAMs were bead-isolated from single-cell suspensions 7 days after treatment. **(D)** TAMs were cultured for 48 hours before their polarization was analyzed. Frequencies of MHC-II^+^ M1 and CD206^+^ M2 TAMs were quantified by flow cytometry. **(E)** Trastuzumab- or E-301–treated TAMs were cocultured with naïve CD8^+^ T cells in the presence of agonistic anti-CD3/28 antibodies, and T cell activation and proliferation were quantified after 48 hours (D and E) (*n* = 9 to 10 replicates). **(F)** Experimental setup for in vitro coculture of primary human CD14^+^ TAMs from LUSC tumors and matched autologous CD8 TILs. TAMs were bead-isolated from single-cell suspensions of primary LUSC tumors and treated with E-301 LOF or E-301. **(G)** Flow cytometric analysis of TAM polarization in T cell cocultures after in vitro E-301 LOF or E-301 treatment. MHC-II^+−^ CD206^−^ M1 and MHC-II^−^CD206^+^ M2 macrophages are shown. **(H)** Proliferation and activation of CD8 TILs after coculture with autologous E-301 LOF− or E-301–treated primary TAMs (*n* = 3). *n* indicates the number of biological replicates. Error bars represent means ± SEM. Statistical analyses were performed using one-way (B) or two-way (D to H) ANOVAs followed by post hoc Šidák corrections for multiple comparisons. *P ≤ 0.05, **P ≤ 0.01, and ***P ≤ 0.001.

We confirmed our findings by flow cytometric immunophenotyping of established B16D5-HER2 tumors, treated with two doses of E-301, E-301 LOF, or trastuzumab (Fig. 5A). We found a higher absolute number of both total CD45^+^ cells and CD8^+^ T cells in tumors treated with E-301 compared with the control-treated tumors (fig. S5A). In agreement with our scRNA-seq results, we further observed an increase in M1-polarized and a decrease in M2-polarized TAMs, evidenced both by major histocompatibility complex (MHC) II and CD206 staining (Fig. 5B) and by the expression of the activation marker CD80 (fig. S5B). Consequently, the M1:M2 ratio was increased upon E-301 treatment (Fig. 5B). Similarly, DCs displayed an increase in the activation marker CD40 (fig. S5C). Last, CD8^+^ T cells showed increased expression of the effector molecules granzyme B and Ki67 (fig. S5D). Similar findings were made in EMT6-HER2 tumors (fig. S5E), with E-301 treatment resulting in a shift from M2- to M1-polarized TAMs (fig. S5F) and an increase in granzyme B– and Ki67-expressing CD8^+^ T cells (fig. S5G). Analysis of macrophages from ICB-treated wild-type and GNE-KO tumors (fig. S5H) revealed a similar repolarization of TAMs in the desialylated tumors, which was further increased by ICB (fig. S5I). Restimulation of NK cells in single-cell suspensions of those tumors showed an increase in the frequency of IFN-γ^+^ NK cells in the GNE-KO tumors compared to the wild-type tumors but no further increase in combination with ICB (fig. S5J). Multiplex analysis of serum cytokine concentrations from E-301–treated mice bearing B16D5-HER2 tumors revealed increased concentrations of cytokines including IL-13, IL-3, IFN-α, IL-6, and IL-27 (fig. S5K).

To further study the cellular mechanism of tumor-targeted desialylation, we used an in vitro coculture of TAMs and T cells. Mice bearing established B16D5-HER2 tumors (500 mm^3^) were treated intraperitoneally with two doses of trastuzumab or E-301, and CD11b^+^ TAMs were bead-isolated 7 days after treatment. After a 48-hour coculture with naïve CD8 T cells, macrophage polarization, as well as T cell proliferation and activation, was measured (Fig. 5C). Treatment with E-301 resulted in an increase in M1-polarized and a decrease in M2-polarized TAMs (Fig. 5D). T cells cocultured with TAMs from E-301–treated tumors were more proliferative and showed increased expression of the activation markers CD25 and granzyme B (Fig. 5E). Similarly, we cocultured bone marrow–derived macrophages (BMDMs) with E-301 LOF– or E-301– treated irradiated B16D5-HER2 tumor cells and proinflammatory macrophage activation measured by intracellular TNF staining (fig. S5L). Coculture with sialylated tumor cells led to a decrease in TNF expression, which was partly abrogated when desialylated tumor cells were used (fig. S5M). In addition, phagocytosis, measured by the macrophage-intrinsic fluorescence intensity of CypHer5E, a pH-sensitive fluorophore, was increased after intraperitoneal injection of desialylated CypHer5E-labeled MC38 GNE-KO tumor cells, compared to labeled wild-type MC38 cells (fig. S5N). This goes in line with previous work and the description of sialic acids as “do not eat me” signals (7) and supports our findings linking tumor sialylation to a protumorigenic TAM polarization.

Last, we wanted to test our findings using primary human LUSC samples. To that end, we isolated CD14^+^ TAMs from single-cell suspensions of primary human LUSC tumors, treated them with E-301 LOF or E-301, and cocultured them with autologous CD8 tumor-infiltrating lymphocytes (TILs; Fig. 5F). Again, desialylation resulted in a shift of TAM polarization toward M1 (Fig. 5G), and CD8 TILs cocultured with desialylated macrophages proliferated more and expressed more CD25 (Fig. 5H). Coculture of monocyte-derived macrophages from healthy peripheral blood mononuclear cells (PBMCs) with autologous CD8 T cells showed a similar result, with desialylation resulting in a more proinflammatory macrophage phenotype and CD8 T cells cocultured with desialylated macrophages being more activated (fig. S5, O to Q). Together, these results demonstrate profound remodeling of the tumor immune microenvironment after therapeutic desialylation by E-301. The specific increase in antitumoral macrophage populations results in an overall shift in intratumoral macrophage polarization and augments the generation of an antitumoral CD8^+^ T cell response.

### Efficacy of tumor-targeted sialidase is dependent on Siglec-E on TAMs

**Fig. 6.**
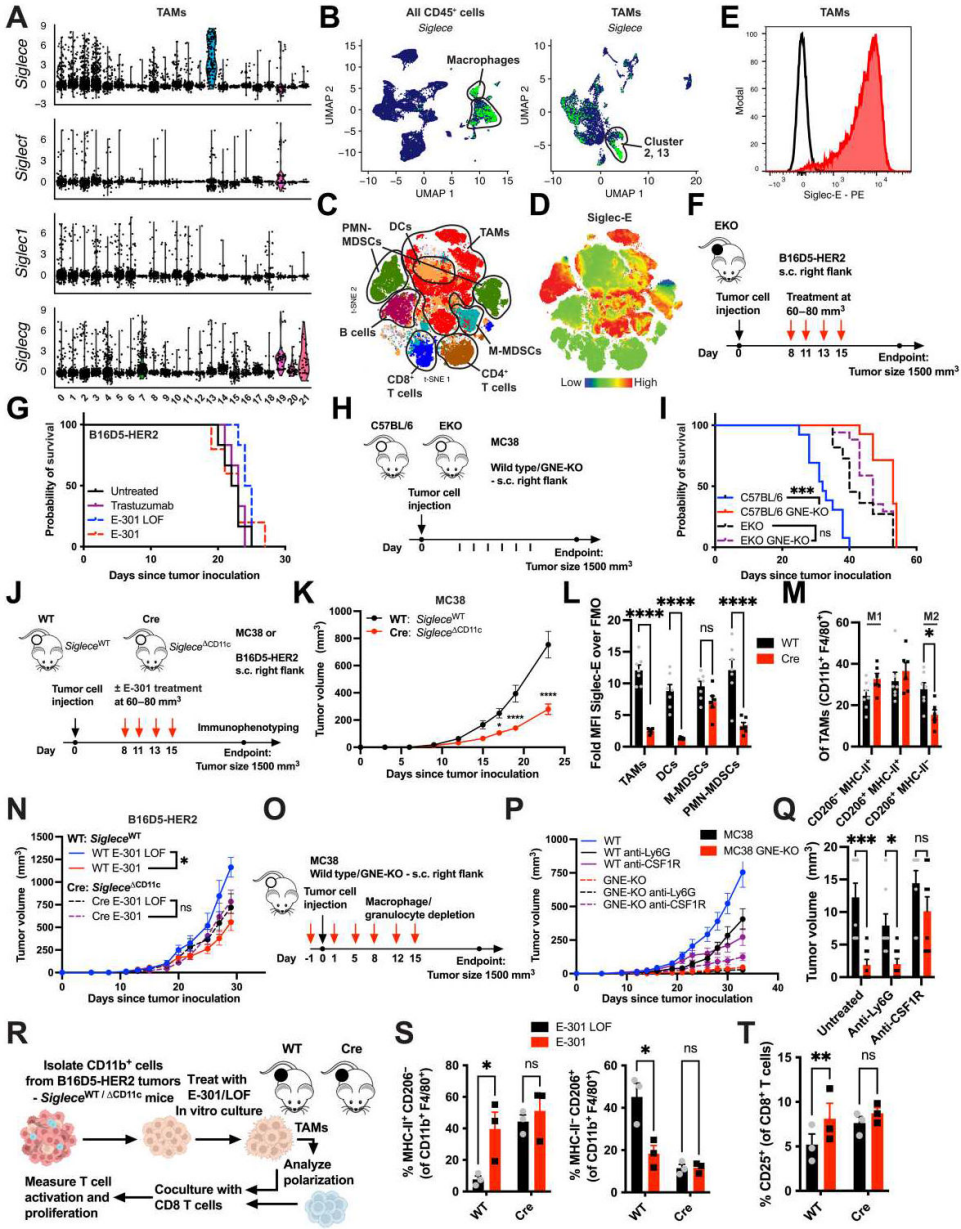
Efficacy of tumor-targeted sialidase is dependent on Siglec-E on TAMs. **(A)** Expression of *Siglece, Siglecf, Siglec1*, and *Siglecg* in all macrophage clusters from scRNA-seq data (Fig. 4E). Sample distribution shown as violin plots. **(B)** UMAP projections of all CD45^+^ cells and all macrophages from scRNA-seq data. Expression of *Siglece* is shown as a color gradient from blue (low) to green (high). **(C)** t-distributed stochastic neighbor embedding (t-SNE) projection of multicolor flow cytometric immunophenotyping of pooled B16D5-HER2 and EMT6-HER2 tumors. Cell populations have been assigned on the basis of marker expression as shown in fig. S6 (A and B). **(D)** Staining intensity for Siglec-E is shown as a color gradient from blue (low) to red (high). **(E)** Representative histogram of Siglec-E staining on CD11b^+^F4/80^+^ TAMs from B16D5-HER2 tumors. Isotype control staining is shown as an empty histogram and anti–Siglec-E staining in red. **(F)** Experimental setup: Mice deficient for Siglec-E (EKO) bearing subcutaneous B16D5-HER2 tumors were treated intraperitoneally with four doses of trastuzumab, E-301 LOF, or E-301 (10 mg/kg) beginning at a tumor size of about 80 mm^3^. **(G)** Survival of EKO mice bearing subcutaneous B16D5-HER2 tumors after trastuzumab, E-301 LOF, or E-301 treatment (*n* = 6 mice per group). **(H)** Experimental setup: C57BL/6 mice and mice lacking Siglec-E (EKO) were subcutaneously injected with wild-type or GNE-KO MC38 tumor cells, and tumor growth was monitored over time. (**I**) Survival of C57BL/6 and EKO mice after subcutaneous injection of MC38 wild-type or GNE-KO tumor cells (*n* = 13 to 17 mice per group). **(J)** Experimental setup: *Siglece*^flox/flox^ mice were crossed to *CD11c*^cre^ mice. Cre-expressing mice (*Siglece*^ΔCD11c^), lacking Siglec-E on all CD11c-expressing cells, were compared to their WT (*Siglece*^WT^) littermate controls. Mice were subcutaneously injected with either MC38 tumor cells and left untreated or with B16D5-HER2 tumor cells in combination with intraperitoneal treatment with four doses of E-301 LOF or E-301 (10 mg/kg) beginning at a tumor size of about 80 mm^3^. **(K)** Average tumor growth of subcutaneously injected MC38 cells in *Siglece*^WT^ and *Siglece*^ΔCD11c^ mice (*n* = 10 to 11 per group). **(L)** Siglec-E expression was measured on different tumor-infiltrating myeloid immune cell types in *Siglece*^ΔCD11c^ mice compared to WT littermate controls by flow cytometry. Siglec-E expression shown as fold change over fluorescence minus one (FMO) control staining. (**M**) Frequencies of CD206^−^MHC-II^+^ (M1), CD206^+^MHC-II^+^, and CD206^+^MHC-II^−^ (M2) macrophages among CD11b^+^F4/80^+^ TAMs. *Siglece*^ΔCD11c^ mice were compared to WT littermate controls (L and M) (*n* = 6 to 7 mice per group). **(N)** Average tumor growth of B16D5-HER2 tumors in *Siglece*^WT^ and *Siglece*^ΔCD11c^ mice treated intraperitoneally with four doses of E-301 LOF or E-301 (*n* = 7 to 9 mice per group). **(O)** Experimental setup for myeloid cell depletion in mice bearing GNE-KO or wild-type MC38 tumors. C57BL/6 mice were treated with either anti-Ly6G antibodies to deplete PMN-MDSCs or anti-CSF1R to deplete TAMs; mice were then injected subcutaneously with wild-type or GNE-KO MC38 tumors cells, and tumor growth was monitored over time. **(P)** Average tumor growth of subcutaneous wild-type or GNE-KO MC38 tumors in mice treated with anti-Ly6G antibodies or anti-CSF1R antibodies. **(Q)** Tumor volumes of wild-type or GNE-KO MC38 tumors after anti-Ly6G or anti-CSF1R treatment on day 12 after tumor cell injection (P and Q) (*n* = 7 to 8 mice per group). **(R)** Experimental setup for the isolation of TAMs from subcutaneous B16D5-HER2 tumors from *Siglece*^ΔCD11c^ or *Siglece*^WT^ mice and coculture with CD8 T cells. **(S)** Macrophages were isolated from B16D5-HER2 tumors from *Siglece*^ΔCD11c^ or *Siglece*^WT^ mice after treatment with E-301 LOF or E-301. Frequencies of macrophages with the indicated phenotypes were quantified by flow cytometry. MHC-II^+^CD206^−^ M1 and MHC-II^−^CD206^+^ M2 polarization was measured. **(T)** CD8 T cell activation was measured after coculture with TAMs from *Siglece*^ΔCD11c^ or *Siglece*^WT^ mice and E-301 treatment (*n* = 3). *n* indicates the number of biological replicates. Error bars represent means ± SEM. Statistical analyses were performed using the log-rank (Mantel-Cox) test or the Gehan-Wilcoxon test, followed by Bonferroni’s correction for multiple comparisons for all survival analyses. Differences between groups were tested using two-way ANOVAs followed by post hoc Šidák corrections for multiple comparisons. *P ≤ 0.05, **P ≤ 0.01, ***P ≤ 0.001, and ****P ≤ 0.0001.

Next, we set out to identify the mechanism by which desialylation affects the polarization of intratumoral macrophages. To that end, we analyzed and compared the expression of the most common inhibitory CD33-related Siglecs, *Siglece, Siglecf*, and *Siglecg*, as well as *Siglec1*, in all TAMs in our scRNA-seq data, finding *Siglece* to be the most prominently expressed (Fig. 6A). Besides being broadly expressed on most TAMs, *Siglece* was most highly expressed on the antitumorigenic TAM cluster 13, which was strongly increased in E-301–treated tumors. This falls in line with previous results, showing it to be the most broadly expressed inhibitory Siglec in mice (4, 26). Further comparison of *Siglece* expression among all CD45^+^ cells confirmed it to be predominantly expressed on macrophages (Fig. 6B). To validate this finding at the protein level and to include granulocytes, which are commonly underrepresented in scRNA-seq, we used multicolor immunophenotyping of single-cell suspensions from both B16D5-HER2 and EMT6-HER2 tumors. T-stochastic next neighbor dimensional reduction of 10 concatenated samples allowed the identification of all major intratumoral immune cell types, including M-MDSCs and PMN-MDSCs (Fig. 6C and fig. S6, A to D). Overlay of the intensity of anti–Siglec-E staining (Fig. 6D and fig. S6E) revealed the strongest expression of Siglec-E on TAMs and PMN-MDSCs (Fig. 6E). Among CD11b^+^ cells, Siglec-E expression coincided with the strongest expression of F4/80 and CD11c expression (fig. S6B).

To validate the role of Siglec-E as the receptor for tumor sialylation, we assessed the growth of subcutaneous B16D5-HER2 tumors in C57BL/6 mice lacking Siglec-E (EKO; Fig. 6F). Treatment of EKO mice bearing B16D5-HER2 tumors with E-301, E-301 LOF, or trastuzumab did not delay tumor growth (Fig. 6G and fig. S6F), supporting the role of Siglec-E in mediating the effects of desialylation. This finding was in accordance with earlier experiments (19). To further corroborate this result, we used MC38 GNE-KO tumor cells as a genetic model of desialylation (Fig. 6H). Again, whereas the growth of MC38 GNE-KO tumors was delayed and survival prolonged in C57BL/6 mice when compared to wild-type MC38 tumors, no difference in tumor growth between MC38 and desialylated MC38 GNE-KO tumors was observed in EKO mice (Fig. 6I and fig. S6, G and H).

In a next step, we wanted to test the effect of TAM-specific loss of Siglec-E expression. To this end, we generated a new conditional KO mouse strain carrying two loxP sites flanking exons 1 to 3 of *Siglece* (*Siglece*^flox/flox^, Elox; fig. S7). Crossing Elox mice with *CD11c*^cre^ mice achieved conditional KO of Siglec-E on all CD11c^+^ cells (*Siglece*^ΔCD11c^). Although CD11c is widely used as a marker for DCs, it is also described as a marker specific for TAMs (27), which we were able to confirm by finding the highest expression of Siglec-E among TAMs to coincide with the highest expression of CD11c (Fig. 6C and fig. S6B). Growth of MC38 tumors was delayed in *Siglece*^ΔCD11c^ mice, lacking Siglec-E on CD11c^+^ cells, compared to their *Siglece*^WT^ control littermates (Fig. 6, J and K). Flow cytometric immunophenotyping of Siglec-E expression on tumor-infiltrating immune cells confirmed the loss of Siglec-E on both DCs and TAMs, as well as on PMN-MDSCs (Fig. 6L and fig. S8A), whereas no difference was detected on lymphoid cells (fig. S8B). Although intratumoral macrophages and DCs showed comparable expression of Siglec-E, splenic macrophages expressed much less Siglec-E than splenic DCs and showed no reduction in Siglec-E expression in *Siglece*^ΔCD11c^ mice, whereas splenic DCs remained affected, further supporting the use of *CD11c*^cre^ as a driver for TAM-specific KO (fig. S8C). Analysis of TAM phenotypes revealed a reduction in M2 polarization in *Siglece*^ΔCD11c^ tumors (Fig. 6M). To address the potential contribution of DCs to the tumor growth delay in the *CD11c*^cre^ model, we crossed Elox mice with *Xcr1*^cre^ mice to obtain a cDC1-specific deletion of Siglec-E. In contrast to the delay observed in *Siglece*^ΔCD11c^ mice, *Siglece*^ΔXcr1^ mice showed no difference in tumor growth compared to their *Siglece*^WT^ littermate controls (fig. S8D).

We then tested whether TAM-specific loss of Siglec-E would affect the antitumor efficacy of therapeutic desialylation. Whereas treatment of *Siglece*^WT^ mice with E-301 again delayed tumor growth, its effect was abrogated in *Siglece*^ΔCD11c^ mice (Fig. 6N and fig. S8E). However, in light of the pleiotropic cellular effects of the *CD11c*^cre^ model, we wanted to further delineate the cellular mechanism underlying the effect of desialylation. To that end, we injected wild-type and GNE-KO MC38 tumor cells into C57BL/6 mice while depleting either Ly6G^+^ granulocytes or colony-stimulating factor 1 receptor 1–positive (CSF1R^+^) TAMs (Fig. 6O). Although both depletions delayed the growth of wild-type MC38 tumors, only anti-CSF1R treatment abrogated the growth delay of GNE-KO MC38 tumors compared to wild-type MC38 tumors (Fig. 6, P and Q). These findings confirm that Siglec-E expression on TAMs is the main mechanistic driver in our tumor model.

Last, we again used an in vitro coculture of TAMs and CD8^+^ T cells. Here, we grew B16D5-HER2 tumors in *Siglece*^WT^ or *Siglece*^ΔCD11c^ mice and bead-isolated CD11b^+^ TAMs. Macrophages were treated with E-301 LOF or E-301 in vitro and cocultured with CD8 T cells from naïve C57BL/6 mice for 48 hours in the presence of agonistic anti-CD3/28 antibodies (Fig. 6R). Treatment of *Siglece*^WT^, but not *Siglece*^ΔCD11c^, TAMs with sialidase shifted macrophage polarization from M2 toward M1 (Fig. 6S) and increased T cell activation (Fig. 6T and fig. S8F).

Together, these experiments identify Siglec-E on TAMs as the receptor for tumor sialylation. Deletion of Siglec-E on all cells or TAMs specifically, as well as depletion of TAMs, delayed tumor growth, enhanced antitumor immunity, and abrogated the effects of both genetic and therapeutic desialylation.

### Targeting tumor sialylation or Siglec-E enhances ICB

**Fig. 7.**
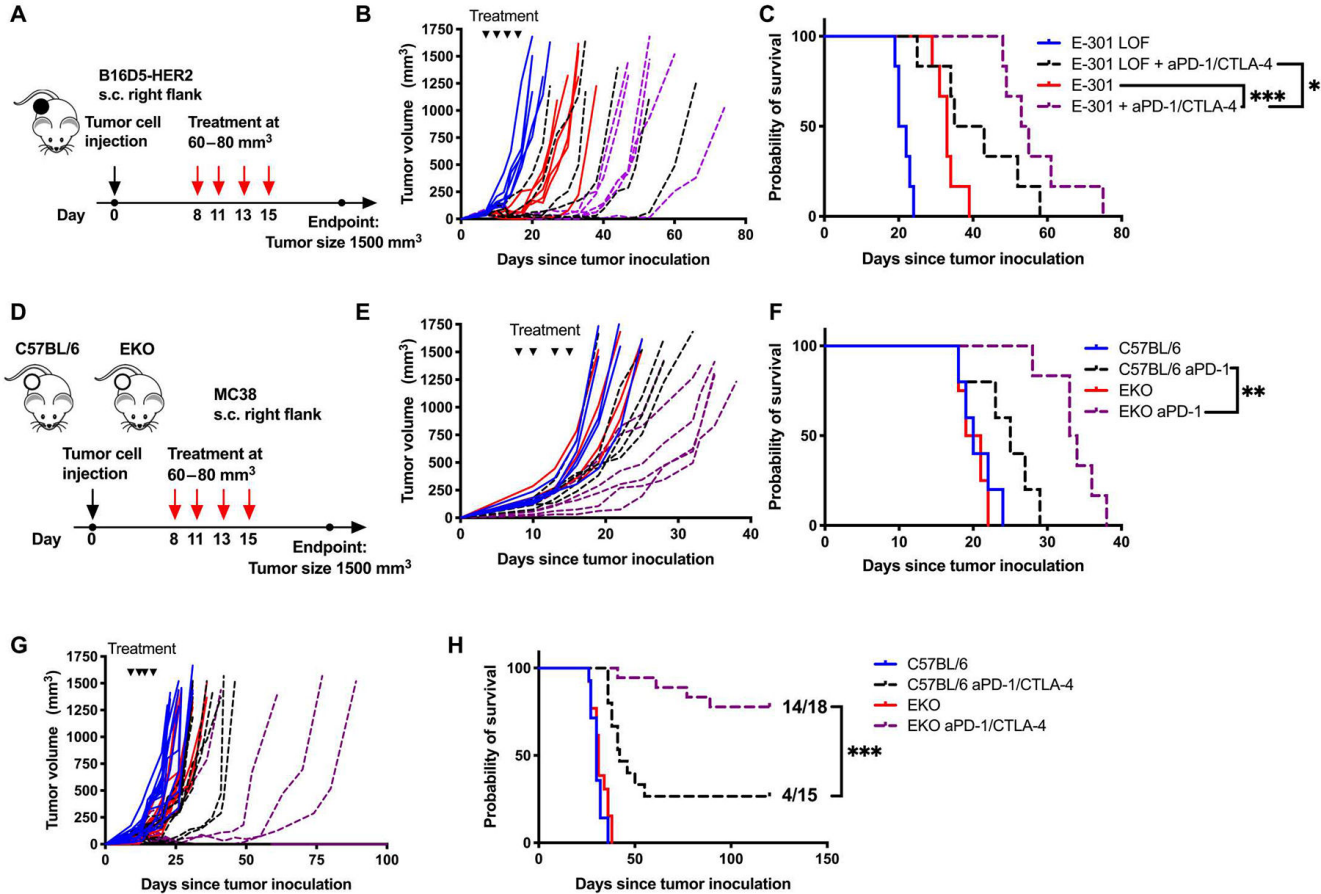
Targeting tumor sialylation or Siglec-E enhances the efficacy of ICB. **(A)** Experimental setup: Mice bearing subcutaneous B16D5-HER2 tumors were treated with four doses of E-301 LOF or E-301, in combination with anti–PD-1/CTLA-4 antibodies (10 mg/kg, i.p.), with the first dose administered once the tumor size reached about 80 mm^3^. **(B)** Growth of individual B16D5-HER2 tumors treated with E-301 LOF or E-301 in combination with anti–PD-1/CTLA-4 antibodies. **(C)** Survival of mice bearing B16D5-HER2 tumors treated with E-301 LOF or E-301 in combination with anti–PD-1/CTLA-4 antibodies (B and C) (*n* = 6 to 8 mice per group). **(D)** Experimental setup for the treatment of wild-type C57BL/6 and EKO mice bearing subcutaneous MC38 tumors with four doses of anti–PD-1 alone or in combination with anti–CTLA-4 intraperitoneally, beginning once the tumor size reached about 80 mm^3^. **(E)** Effect of anti–PD-1 ICB on the growth of individual MC38 tumors in wild-type C57BL/6 and EKO mice. **(F)** Survival of wild-type C57BL/6 and EKO mice bearing MC38 tumors after treatment with PD-1 blockade (E and F) (*n* = 4 to 6 mice per group). **(G)** Effect of combined anti–PD-1 and anti–CTLA-4 ICB on the growth of individual MC38 tumors in wild-type C57BL/6 and EKO mice. **(H)** Survival of wild-type C57BL/6 and EKO mice bearing MC38 tumors after treatment with PD-1 and CTLA-4 blockade (G and H) (*n* = 13 to 18 mice per group). *n* indicates the number of biological replicates. Statistical analyses were performed using the Gehan-Wilcoxon test followed by Bonferroni’s correction for multiple comparisons for all survival analyses. *P ≤ 0.05, **P ≤ 0.01, and ***P ≤ 0.001.

We then asked whether tumor-targeted desialylation could be combined with PD-1 and CTLA-4 blockade in the B16D5-HER2 model (Fig. 7A). Combination of E-301 with PD-1– and CTLA-4–blocking antibodies augmented the inhibition of tumor growth and delayed time to progression compared with both E-301 and ICB in combination with E-301 LOF (Fig. 7, B and C). This finding corroborated the benefits of combining desialylation and ICB, which we had observed using several genetic models of desialylation (Fig. 1, H to J). Together, these data clearly demonstrate an enhancing effect of targeting tumor sialylation and PD-1/CTLA-4 blockade.

On the basis of our finding that Siglec-E mediates the effect of therapeutic desialylation, we wanted to test whether the loss of Siglec-E would similarly improve ICB efficacy. To that end, we inoculated MC38 tumors in both wild-type C57BL/6 and EKO mice (Fig. 7D). PD-1 blockade was more efficacious in delaying tumor growth and prolonging survival in EKO mice than in wild-type C57BL/6 mice (Fig. 7, E and F). This effect was further enhanced when a combination of PD-1 and CTLA-4 blockade was used, with 14 of 18 (78%) EKO mice rejecting their tumors, compared to 4 of 15 (27%) wild-type mice (Fig. 7, G and H). These findings further support the sialoglycan-Siglec axis as a target for immunotherapy, as both genetic and therapeutic desialylation and loss of Siglec-E enhances ICB.

## DISCUSSION

Because tumor sialylation dampens antitumor immune responses, targeting the sialoglycan-Siglec axis has been proposed as a new immunotherapeutic approach. Our data provide mechanistic evidence for tumor sialylation–mediated immune suppression, demonstrate the efficacy and feasibility of therapeutic desialylation, and highlight its potential for use in combination with classical ICB. We show that inhibitory Siglec-E on TAMs is the main receptor for cancer-associated sialic acids and demonstrate specific antitumoral repolarization of TAMs upon tumor desialylation or loss of Siglec-E (fig. S9).

Specifically, we demonstrated that genetic and therapeutic desialylation of tumor cells delayed tumor growth, enhanced ICB, and resulted in an antitumorigenic polarization of TAMs. These findings confirm previous reports using a range of tumor models and experimental approaches (5, 17, 19, 28). Loss of Siglec-E abrogated the efficacy of therapeutic and genetic desialylation, and KO of Siglec-E on TAMs was sufficient to delay tumor growth and repolarize macrophages. This confirms and expands on work showing increased tumor growth and protumorigenic M2 macrophage polarization in tumors overexpressing a sialyltransferase. Mirroring our results, the effect was abrogated in *Siglece*^−/−^ animals (12, 13). Desialylation of TAMs from primary human LUSC tumors resulted in a shift toward M1 polarization and increased T cell activation, corroborating data from human TAMs, showing a similar sialylation-dependent M2 polarization, which is mediated by human Siglec-7 and Siglec-9 and reversible by desialylation (9-11). In addition, we observed enhanced phagocytosis of desialylated tumor cells by macrophages, in line with previous work demonstrating sialidase treatment to increase phagocytosis of lymphocytes and the recent (29) description of human Siglec-10 as a receptor for do not eat me signals in cancer (7). Similarly, Siglec-2 (CD22) has been shown to mediate sialic acid–induced inhibition of microglial phagocytosis (30). At this time, it remains unclear whether either mechanism predominates in our models or whether other factors such as direct TAM–T cell interactions in the tumor microenvironment might be at play (31).

Other work using desialylated tumor cell lines or intratumoral administration of a fluorinated sialic acid mimetic, blocking de novo biosynthesis of sialoglycans, revealed similar antitumor immunity–promoting effects (5, 17, 28). The uptake of sialylated antigens by DCs was shown to enhance the induction of T_regs_ through engagement of Siglec-E, which might reflect the interactions between DCs and naïve CD4^+^ T cells in the hypersialylated tumor microenvironment (18, 32). We observe a reduction in the frequency of naïve CD4^+^ cells upon E-301 treatment (fig. S4F). We recently described the up-regulation of human Siglec-9 and murine Siglec-E on tumor-infiltrating T cells in humans and mice (5, 16). This up-regulated expression dampens T cell activation in the presence of Siglec-ligands, which can be reversed by blockade of sialoglycan binding. It is conceivable that direct reinvigoration of T cell effector function in the tumor microenvironment might contribute to the results obtained in this work, as has been shown for N-glycan branching and chimeric antigen receptor (CAR) T cell function (33). Furthermore, a recent screen for surface proteins inhibiting T cell activation led to the identification of Siglec-15 as a potential target for cancer immunotherapy, which could be influenced by sialylation (34).

Besides binding to Siglecs, sialic acid–containing glycans could also inhibit adaptive immunity by acting as alternative ligands for CD28 and inhibiting CD28-CD80 interactions (35). In addition, sialylation can affect the stability of receptors (36), such as epidermal growth factor receptor 1, where it modulates signaling by mediating dimerization (37). Desialylation could further enhance the formation of galectin lattices by exposing underlying galactose residues (38). It is therefore possible that the effects of tumor desialylation extend beyond those mediated by Siglec receptors. However, our data point to Siglec-E–expressing TAMs being a main molecular and cellular mediator of desialylation of the tumor microenvironment. Combined, these findings suggest a clear role for tumor sialylation in dampening antitumor immune responses, both through direct action on T cells and indirectly by affecting the phenotypes of other tumor-infiltrating immune cells, such as DCs and TAMs.

Therapeutic targeting of the sialoglycan-Siglec axis can be achieved by either targeting of the sialic acid–containing ligands or the Siglec receptors. Although the latter can be accomplished using blocking antibodies (8), functional redundancy and potential compensation among the human Siglec repertoire might ultimately dampen the efficacy of such an approach. Therefore, direct targeting of tumor sialylation might prove a more effective option (19). Blocking tumor hypersialylation with chemical inhibitors of sialic acid biosynthesis results in pronounced desialylation, increased T cell infiltration, and CD8^+^ T cell–dependent delay in tumor growth (28). However, systemic application in mice leads to strong desialylation of all tissues, lasting up to several weeks, and is ultimately fatal, limiting the applicability of this approach to localized intratumoral applications (39). In addition, complete abrogation of tumor sialylation has been shown to induce apoptosis in CD8^+^ T cells, which might limit the efficacy of such approaches (40). In contrast, mice systemically injected with *Vibrio cholerae* sialidase only show transient toxicity, reflecting the short-lived nature of enzymatic desialylation (41). We demonstrate further reduced toxicity by targeting sialidase to tumor antigens, thereby restricting the sialidase activity to the tumor microenvironment. We suggest this approach to be favorable both in terms of antitumor efficacy and potential toxicity.

A limitation of our study is the requirement of a tumor antigen as the target for therapeutic desialylation. Here, we used HER2 as a well-studied cancer-associated antigen. Although HER2 targeting is effective and has improved the prognosis of patients with HER2-positive breast and gastric cancer (42), many cancer types lack a selectively and consistently expressed target. Additional work will be needed to specify the requirements for cancer-associated targets for therapeutic desialylation. The relative contributions of the desialylation of tumor cells and immune cells to the observed antitumor activity remain undefined. It remains unclear whether the desialylation of tumor cells is an essential prerequisite for the effectiveness of therapeutic desialylation or whether targeting of intratumoral immune cells might elicit comparable effects. This could open the possibility of constructing antibody-sialidase constructs against antigens expressed on intratumoral immune cells, expanding the range of potential cancer types that may be targeted.

Our work demonstrates that therapeutic targeting of tumor sialylation is effective in vivo and enhances the efficacy of PD-1 and CTLA-4 blockade. Using scRNA-seq, we showed mechanistically how therapeutic desialylation repolarizes TAMs toward an antitumorigenic phenotype and augments the adaptive antitumor immune response. We identified inhibitory Siglec-E on TAMs as the main target of desialylation. Together, these results provide a strong rationale for the further clinical development of sialoglycan-Siglec targeting agents and their combination with PD-1– and CTLA-4–blocking immunotherapies.

## MATERIALS AND METHODS

### Study design

The purpose of this study was to evaluate the efficacy of therapeutic desialylation and to investigate the underlying mechanism of action. Mice bearing HER2-expressing tumors were treated with sialidase-trastuzumab fusion proteins, and tumor sizes were monitored over time. Where specified, tumors were harvested, and their immune compositions were analyzed by flow cytometry. Mice that developed ulcerations were excluded from experiments. All experiments were randomized and blinded where possible. Sample sizes were determined on the basis of expected effect sizes from pilot experiments. In general, group sizes of five or more mice were used. Differences in tumor growth were tested using two-way analyses of variance (ANOVAs), correcting for multiple measurements.

### Mice

All mouse experiments were approved by the local ethics committee (approval 2747, Basel Stadt, Switzerland) and performed in accordance with the Swiss federal regulations. C57BL/6 and BALB/c mice were obtained from Janvier Labs (France) and bred in-house at the Department of Biomedicine, University Hospital Basel, Switzerland. The full-body Siglec-E–deficient mouse strain (EKO) was received from A. Varki (University of California, San Diego) and had been previously described (43). EKO mice were bred in-house and backcrossed to the local C57BL/6 strain in heterozygous pairings for more than nine generations. *Siglece*^flox/flox^ mice were generated by Biocytogen as previously described (32). *Itgax*^cre^ (CD11c^cre^) mice were provided by D. Finke (University of Basel, Switzerland). *Xcr1*^cre^ mice were a gift from T. Kaisho (Wakayama Medical University, Japan) (44). All animals were housed under specific pathogen–free conditions.

### Cells and cell culture

All cell lines were maintained in Dulbecco’s modified Eagle’s medium, supplemented with 10% heat-inactivated fetal bovine serum (FBS; PAA Laboratories), 2 mM l-glutamine, 1 mM sodium pyruvate, streptomycin (100 μg/ml), and penicillin (100 U/ml; Gibco). The B16D5-HER2, EMT6-HER2, EMT6-HER2 GNE-KO, and MC38 GNE-KO cell lines have been described previously (5, 45). The parental B16F10 cell line was obtained from the American Type Culture Collection, and the generation of B16F10 GNE-KO cells is described below. All cells were cultured at 37°C under 5% CO_2_ atmosphere and cultured for a minimum of three passages before being used.

### Tumor models

For tumor experiments, 7- to 11-week-old mice were used. Sex-matched wild-type littermates were used as controls for all experiments involving transgenic mice. Experiments were approved by the local ethical committee (BS, cantonal number 3036). Tumor cell injections were performed as described previously (45). For the subcutaneous MC38 and B16 models (wild-type, GNE-KO, and HER2 expressing), mice were subcutaneously injected with 500,000 tumor cells in phosphate-buffered saline (PBS) in their right flank or, in some cases, in both the right and left flanks. For the orthotopic EMT6 model, mice were injected with 1,000,000 EMT6 cells in PBS (wild-type, GNE-KO, and HER2 expressing) in the right or left mammary fat pad of female BALB/c mice. Tumor size and overall health score, as well as body weights in some experiments, were measured and monitored three times per week. Perpendicular tumor diameters were measured using a caliper, and tumor volume was calculated according to the following formula: tumor volume (in mm^3^) = (*d*^2^ × *D*)/2, where *d* and *D* are the shortest and longest diameters (in mm) of the tumor, respectively. Mice were euthanized before the size of their tumors reached 1500 mm^3^. Animals that developed ulcerated tumors were euthanized and excluded from further analysis.

### In vivo treatments

Antibodies for in vivo depletion of CD8^+^ T cells (anti-mouse CD8a, 53-6.7), macrophages (anti-mouse CSF1R, AF598), and granulocytes (anti-mouse Ly6G, 1A8 and anti-rat kappa light chain, MAR 18.5), as well as in vivo immune checkpoint inhibitor (ICI) treatment (anti-mouse PD-1, RMP1-14 and anti-mouse CTLA-4, 9D9), were purchased from Bio X Cell. For T cell depletion, anti-CD8 depleting antibody was administered intraperitoneally at 10 mg/kg on days −2 and 0 relative to the time of tumor cell injection, and depletion was maintained by weekly treatments throughout the duration of the experiment. Depletion of macrophages was achieved by intraperitoneal administration of anti-CSF1R antibody at 400 μg per mouse twice per week, beginning on days −1 and +1 relative to the time of tumor cell injection. Granulocytes were depleted by a combination of rat anti-mouse Ly6G and anti-rat kappa light chain antibodies, both at 100 μg per mouse twice per week, following the same schedule used for macrophage depletion (46). For ICB experiments, treatments were administered intraperitoneally at 10 mg/kg once the tumors reached an average size of 80 to 100 mm^3^, and a total of four doses were given every second to third day, unless specified otherwise. For 7-day treatments, tumors were allowed to grow until they reached a size of about 500 mm^3^, and they were then treated intraperitoneally at 10 mg/kg for a total of two doses.

### Antibody-sialidase constructs

The sequences encoding for E-301 and E-301-LOF containing *Salmonella typhimurium* sialidase were cloned into the mammalian expression vector pCEP4 (Thermo Fisher Scientific). Human embryonic kidney 293T cells were then transiently transfected with the DNA constructs using the Expi293 system and following standard protocols according to the manufacturer’s instructions (Thermo Fisher Scientific). Purification of E-301 and E-301 LOF was performed directly from transfection harvests using a HiTrap Protein A affinity column (GE Healthcare) and eluted with 1 M arginine (pH 3.9). Anion-exchange chromatography was used as a secondary purification method, and the final product was dialyzed into PBS (pH 7.4). The biochemical characterization of E-301 and E-301 LOF, including purity, HER2 binding affinity, and enzymatic activity, was conducted as described previously (25).

### Generation of B16F10 GNE-KO tumor cells

KO of *Gne* in B16F10 tumor cells was performed using clustered regularly interspaced short palindromic repeats (CRISPR)–Cas9–mediated gene editing. Guide RNAs were designed online using E-CRISP (e-crisp.org), synthesized by Microsynth, and cloned into the pSpCas9(BB)-2A-GFP (green fluorescent protein) (PX458) vector, which is a gift from F. Zhang (Massachusetts Institute of Technology; Addgene plasmid #48138; http://n2t.net/addgene:48138; RRID: Addgene_48138) (47). The parental cell line was transiently transfected, and single GFP^+^ cells were sorted into 96-well plates. After their recovery and expansion, individual clones were screened for cell surface sialylation by lectin staining, and candidates were confirmed to be GNE-KO. Multiple GNE-KO clones were selected and pooled to avoid clonal effects. The wild-type parental cell line and transfected and sorted clones showing normal cell surface sialylation were used as controls. Cell surface sialylation was analyzed by flow cytometry using lectin staining.

### Tumor digests

Primary tumor samples were collected during surgery. Sample collections were approved by the local ethical committee [Ethikkom-mission Nordwestschweiz (EKNZ) 2018-01990]. For the preparation of single-cell suspensions from both human and mouse tumors, tumors were collected, and surgical specimens were mechanically dissociated and subsequently digested using Accutase (PAA Laboratories), collagenase IV (Worthington), hyaluronidase (Sigma-Aldrich), and deoxyribonuclease type IV (Sigma-Aldrich) for 1 hour at 37°C under constant agitation. Cell suspensions were filtered through a 70-μm mesh, and for the analysis of tumor-infiltrating immune cells, CD45^+^ cells were further enriched by Histopaque-1119 density gradient centrifugation (Sigma-Aldrich). Splenocytes were isolated by mechanical disruption using the end of a 1-ml syringe, filtration through a 70-μm mesh, and lysis of red blood cells using red blood cell lysis buffer (eBioscience). Samples were frozen in 90% FBS and 10% dimethyl sulfoxide and stored in liquid nitrogen until the time of analysis.

### Multicolor flow cytometry

Flow cytometry was performed on single-cell suspensions of cell lines, blood samples, splenocytes, and tumor digests with the antibodies listed in tables S1 and S2. To prevent unspecific staining, cells were initially blocked with anti-mouse Fcγ III/II receptor (CD16/CD32) antibodies, and dead cells were excluded by staining with a fixable live/dead cell exclusion dye (BioLegend). Then, cell suspensions were stained for cell surface antigens with primary fluorophore-conjugated antibodies for 20 min at 4°C in fluorescence-activated cell sorting (FACS) buffer (PBS, 2% FBS, and 0.5 mM EDTA). Stained samples were fixed with IC fixation buffer (eBioscience) until the time of analysis. For intracellular antigens (IL-2, IFN-γ, TNF, FoxP3, and Ki67), cells were first stained against cell surface antigens, fixed, and permeabilized (eBioscience) followed by staining with antibodies directed against intracellular antigens. For intracellular cytokine staining, single-cell suspensions of tumor digests were restimulated ex vivo with phorbol 12-myristate 13-acetate (20 ng/ml) and ionomycin (500 μg/ml) for 6 hours, and brefeldin A (BD Pharmingen) was added for the last 4 hours. All samples were acquired on a LSRII Fortessa flow cytometer (BD Biosciences) or Cytek Aurora (Cytek Biosciences) and analyzed using FlowJo 10.3 (TreeStar Inc.) after sequential doublet exclusion [forward scatter (FSC)–area (A) versus FSC-height (H) and side scatter (SSC)–A versus SSC-W] and live/dead cell discrimination.

### Lectin staining

For analysis of lectin binding by immunofluorescence, frozen sections of optimal cutting temperature compound–embedded tumors were cut and prepared using a cryostat. Biotinylated PNA, MAL II, SNA, and ConA lectins (Vector Laboratories) were used at 10 μg/ml and incubated in FACS buffer for 20 min at 4°C. Binding of lectins for microscopy was then detected by incubation with Streptavidin-Cy3, quantified, and normalized to the respective area of 4′,6-diamindino-2-phenylindole staining. For flow cytometric analysis of lectin binding, single-cell suspensions of tumor digests were blocked, live/dead stained, and incubated with biotinylated lectins at 10 μg/ml. After detection using streptavidin–phycoerythrin (PE)–Cy7, samples were fixed (IC fixation buffer, eBioscience) and acquired on a CytoFLEX flow cytometer (Beckman Coulter). Lectin staining was quantified after doublet and live/dead exclusion using the geometric mean fluorescence intensity. ConA was used as a sialic acid–independent control. A direct staining with PNA-PE (GeneTex) was used to assess desialylation of intratumoral immune cells.

### Multiplex cytokine measurements

Cytokine concentrations in the serum of B16D5-HER2–bearing C57BL/6 mice were measured 7 days after treatment with E-301, E-301 LOF, and trastuzumab. Blood was collected retro-orbitally at the time of euthanasia and clotted for a minimum of 30 min in Microvette 200 Z-Gel tubes containing a clotting activator (Sarstedt). Clotted samples were centrifuged at 10,000*g* for 10 min at room temperature and frozen at −80°C. Measurement of cytokine concentrations was performed using the Luminex Cytokine & Chemokine 36-Plex Mouse ProcartaPlex Panel 1A kit (Thermo Fisher Scientific). Statistical significance was determined using multiple unpaired *t* tests with post hoc Bonferroni’s correction for multiple comparisons.

### Generation of sialylation gene expression signatures from TCGA data

First, a set of genes involved in sialic acid biosynthesis and metabolism was generated by merging genes from the Reactome gene set “sialic acid metabolism” (ID R-HSA-4085001) and the Gene Ontology gene set “sialylation” (ID GO:0097503). The merged gene set contained the following genes: *ST3GAL3, ST6GALNAC5, ST6GAL-NAC3, ST3GAL5, ST6GAL2, ST3GAL6, ST6GAL1, ST8SIA4, ST3GAL1, ST6GALNAC4, ST6GALNAC6, ST8SIA6, ST3GAL4, ST8SIA1, ST8SIA2, ST3GAL2, ST6GALNAC2, ST6GALNAC1, ST8SIA5, ST8SIA3, C20orf173, NPL, NEU2, NEU4, GLB1, NEU1, SLC17A5, SLC35A1, GNE, NANS, NEU3, CMAS, NANP*, and *CTSA*. The immune gene set used was described previously and contains 3021 immune-related genes (22). The fragments per kilo-base of transcript per million mapped reads upper quartile (FPKM-UQ) gene expression values of the TCGA database were retrieved with TCGAbiolinks (48). The log_2_ FPKM-UQ expression of each sialylation-associated gene was Pearson correlated with the expression of each immune-related gene using the solid cancers from the TCGA dataset. The correlation begin{bmatrix} was used as input for *k*-means clusterings using *k* values ranging from 1 to 10. The clustering was done with the kmeans function in R using the following settings: nstart = 50 and iter.max = 15. The elbow method indicated that *k* = 5 minimized total intracluster variation; hence, a total of five sialylation clusters (gene sets) were kept for further analyses. Median expression values of each gene set were used for statistical analyses.

### Survival analysis of TCGA data

For each cancer type, the median of the expression values of the genes in each gene set was calculated. Patient data were divided into quartiles based on their expression values (low, intermediate-low, intermediate-high, and high). Survival analysis was performed for all five clusters.

### Correlation with immune cell proportions, immune gene sets from Reactome, and T cell dysfunction and exhaustion scores

The median log_2_ FPKM-UQ expression of gene set 1 was Pearson correlated with the proportion of 10 immune cell types. The proportions of immune cells were adopted from The Cancer Immunome Atlas database (https://tcia.at/cellTypeFractions). The proportions used were retrieved using the method “quanTIseq lsei TIL10.” Relevant immune gene sets were selected from the Reactome database. The median log_2_ FPKM-UQ expression of each sialylation gene of a gene set was Pearson correlated with each gene from the respective immune gene set. The mean of all absolute correlation coefficients was calculated to obtain a correlation score. The median log_2_ FPKM-UQ gene set 1 expression from the TCGA LUSC and KIRC datasets was Pearson correlated with published T cell dysfunction and exclusion scores (24).

### Tissue microarray analysis

The tissue microarray HKid-CRCC150CS-01, containing 75 cores of KIRC tumors with adjacent control tissue and survival information, was obtained from Biomax. Patient characteristics including age, sex, grade, stage, and outcome are publicly available at www.biomax.us/tissue-arrays/Kidney/HKid-CRCC150CS-01. Slides were deparaffinized and stained with a hexameric Siglec-9 Fc construct (HYDRA, Palleon Pharmaceuticals) (23). Binding was detected and visualized by immunohistochemistry; slides were scanned, and staining intensities were quantified using Fiji (ImageJ). Patients were linearly divided into tertiles based on their staining intensities, and survival analysis was performed.

### Patient samples

The local ethics committee in Basel, Switzerland approved the sample collection and the use of the corresponding clinical data (EKNZ, Basel Stadt, Switzerland). Informed consent was obtained from all patients before sample collection. Tumor samples were collected locally at the thoracic surgery of the University Hospital Basel, digested and processed as described above, and frozen as single-cell suspensions.

### Human tumor-infiltrating T cell stimulation

Single-cell suspensions of human tumor digests were prepared as described above, thawed, and seeded in RPMI 1640 medium supplemented with 10% heat-inactivated FBS (PAA Laboratories), 2 mM l-glutamine, 1 mM sodium pyruvate, streptomycin (100 μg/ml), and penicillin (100 U/ml) (all Gibco). The cells were then treated with *V. cholerae* neuraminidase (10 mU/ml; Roche) for 48 hours. Supernatants were frozen at −80°C, and the cells were stained for markers of T cell activation.

### Generation of conditional Siglec-E KO mice

Conditional *Siglece*^flox/flox^ C57BL/6 mice (Elox mice) were generated by the introduction of two loxP sites flanking exons 1 to 3 of *Siglece* using CRISPR-Cas9 (fig. S7). Specific deletion of Siglec-E in TAMs was achieved by breeding *Siglece*^flox/flox^ mice with *Itgax*^cre^ mice. Cre-negative littermates were used as controls. Conditional KO was confirmed by flow cytometry (Fig. 6L).

### Analysis of scRNA-seq data

Samples were demultiplexed and aligned using Cell Ranger 6.1 (10X Genomics) to genome build GRCm38 to obtain a raw read count begin{bmatrix} of barcodes corresponding to cells and features corresponding to detected genes. High-quality sequenced libraries had comparable technical characteristics. Read count matrices were processed, analyzed, and visualized in R v.4.0.0 (R Core Team, 2013) using Seurat v.4 (49) with default parameters in all functions, unless specified. Cells with more than 200 detected genes were initially retained, and then those with greater than 10% of mitochondrial genes as a fraction of all detected genes were removed from the analysis. These latter low-quality cells also displayed low total unique molecular identifier counts. Filtered samples were normalized using a regularized negative binomial regression (SCTransform) (50) and integrated with the reciprocal principal components analysis approach followed by mutual nearest neighbors, using 50 principal components. Integrated gene expression matrices were visualized with a uniform manifold approximation and projection (UMAP) (51) as a dimensionality reduction approach. Resolution for cell clustering was determined by evaluating hierarchical clustering trees at a range of resolutions (0 to 1.2) with Clustree (52), selecting a value inducing minimal cluster instability. Datasets were subset to include only specific cells based on gene expression. Subset datasets were then split along conditions and processed anew as described above.

Differentially expressed genes between clusters were identified as those expressed in at least 25% of cells with a greater than 0.3 natural log fold change and an adjusted *P* value of less than 0.01, using the FindMarkers function in Seurat v.4 with all other parameters set to default. The dataset was uploaded to Gene Expression Omnibus (accession number GSE208133).

### Mouse coculture model

To study macrophage–T cell interactions, mice bearing established B16D5-HER2 tumors were treated with two injections of trastuzumab or E-301 (10 mg/kg) when tumors reached about 500 mm^3^. CD11b^+^ intratumoral macrophages were bead-isolated using the EasySep positive selection kit (STEMCELL Technologies). In some experiments, Siglece^ΔCD11c^ mice were used to generate TAMs lacking Siglec-E expression. CD8^+^ T cells were bead-isolated with an EasySep positive selection kit (STEMCELL Technologies) from the spleens of naïve C57BL/6 mice and T cells labeled with CellTrace Violet per the manufacturer’s instructions (Invitrogen). Isolated TAMs were cultured overnight, and attached cells were analyzed for their polarization or cocultured for 48 hours at a 1:5 ratio with CD8^+^ T cells, in the presence of agonistic anti-CD3 (2.5 μg/ml; BioLegend) and anti-CD28 (5 μg/ml; BD Biosciences) antibodies (table S3), at which point T cell activation was analyzed. TAMs from Siglece^ΔCD11c^ mice were treated with E-301 or E-301 LOF during their overnight culture, as well as during the coculture with CD8^+^ T cells.

BMDMs were generated by plating bone marrow cells freshly isolated from tibias and femurs into 10-cm dishes. Cells were maintained in RPMI 1640 medium (Sigma-Aldrich) supplemented with 10% heat-inactivated FBS (PAA Laboratories), 1 mM sodium pyruvate (Gibco), 1× MEM Non-essential Amino Acid Solution (Sigma-Aldrich), streptomycin (100 μg/ml), penicillin (100 U/ml; Gibco), 0.05 mM 2-mercaptoethanol (Gibco), and mouse macrophage colony-stimulating factor (M-CSF; 10 ng/ml; PeproTech) for 5 days. BMDMs were incubated with lipopolysaccharide (40 ng/ml) and IFN-γ (100 ng/ml) or IL-4 (20 ng/ml) and irradiated B16D5-HER2 tumor cells (5 grays) for 24 hours at a 1:2 ratio in the presence of E-301 or E-301 LOF.

### Human coculture model

For the coculture of human monocyte-derived macrophages (momacs), human PBMCs were isolated by density gradient centrifugation using Histopaque-1077 (Sigma-Aldrich) from buffy coats obtained from healthy blood donors (Blood Bank, University Hospital Basel, Switzerland). Macrophages were differentiated by culturing adherent monocytes with human M-CSF (10 ng/ml; PeproTech) for 7 days. Autologous human T cells were isolated from frozen PBMCs of matched samples using the EasySep Human T cell isolation kit (STEMCELL Technologies) according to the manufacturer’s instructions. T cells were stained with CellTrace Violet per the manufacturer’s instructions (Invitrogen) for 20 min at 37°C, and cells were washed twice with complete medium. T cells were cultured either alone or cocultured with mo-macs (1:5 ratio), and T cell activation and proliferation were induced by ImmunoCult Human, anti-CD3/CD28/CD2 T cell activator (25 μl/ml; STEMCELL Technologies; table S3), and IL-2 (100 IU/ml; Proleukin).

In the case of primary LUSC tumors, CD14^+^ TAMs were bead-isolated using an EasySep positive selection kit (STEMCELL Technologies), cultured overnight in the presence of E-301 or E-301 LOF to adhere, washed, and analyzed for macrophage polarization. Adherent TAMs were cocultured with autologous CD8^+^ TILs (1:5 ratio) for 5 days in the presence of E-301 LOF or E-301. T cells were labeled with CellTrace Violet per the manufacturer’s instructions, and proliferation was induced by ImmunoCult Human anti-CD3/CD28/CD-2 cell activator (25 μl/ml; STEMCELL Technologies) and IL-2 (100 IU/ml; Proleukin).

### Glycan extraction and MS

The enzymatic release, extraction, reduction, and purification of glycans were as described previously with slight modifications (53, 54). Briefly, confluent cells were harvested with a non-enzymatic method, such as scraping from plates using cell dissociation buffer (13151014, Gibco) for 5 to 10 min. The cell pellet was then washed three times with PBS. The cells were then lysed using radio-immunoprecipitation assay (RIPA) buffer (Genesearch; catalog no. 9806S) in the presence of complete protease inhibitor cocktail (CO-RO, Roche or 11697498001, Merck). A volume of 500 μl of RIPA buffer was added to each tube and left at 4°C overnight with periodic gentle shaking. The samples were then sonicated for 30 min in a sonication bath. The cell lysis was then centrifuged at 4°C for 10 min at 16,000*g*, and the supernatant was collected in a new microfuge tube. The supernatant containing the cell lysate was subjected to chlorform:methanol:water (1:2:2) extraction to separate the lipids, glycolipids, and proteins. Samples were briefly vortexed and then incubated on ice for 1 hour. They were then centrifuged for 20 min at 10,000 rpm. The aqueous top layer was removed followed by the addition of two volumes of methanol to precipitate the proteins. The precipitated protein pellet was resuspended in 200 μl of 4 M urea and quantified using a bicinchoninic acid assay. Protein lysate (20 μg) from each sample and replicates (*n* = 3) were dot blotted onto an ethanol-wetted polyvinylidene fluoride (PVDF) membrane and dried overnight at room temperature. The PVDF membrane was then washed in methanol for 15 min at room temperature with shaking, followed by water for 15 min at room temperature with shaking. To visualize the immobilized protein, the membrane was stained with direct blue staining solution and then destained. Individual sample spots 6 mm in diameter were excised from the PVDF membrane and submerged into wells containing 100 μl of 1% polyvinylpyrrolidon (PVP) 40, where they were incubated for 5 min, and then washed thrice with 200 μl of water. *N*-linked glycans were enzymatically released using peptide *N*-glycosidase F (2 μl of 1000 U; New England Biolabs), and 28 μl of water was added to each well and incubated overnight at 37°C. The wells were then rinsed with 50 μl of water, and the solutions were collected in individual microfuge tubes. The samples were then acidified with 10 μl of 100 mM ammonium acetate (pH 5) and incubated for an additional 1 hour at room temperature. Released *N*-glycans were reduced under alkaline conditions by the addition of 20 μl of 1.25 M NaBH_4_ in 100 mM KOH and incubated for 3 hours at 50°C. After cooling to room temperature, the reaction was neutralized with 2 μl of glacial acetic acid.

Sodium salts of *N*-glycan solutions were removed by passage through and subsequent washing of cation exchange microcolumns constituted by packing 50 μl of Dowex 50W X8 (Sigma-Aldrich) in a ZipTip C18 tip (Merck Millipore), activated with 50 μl of 1 M HCl, followed by washing with methanol and water before addition of the sample. Reduced sample solutions were passed through the prepared columns using a bench top microcentrifuge at full speed for 15 to 30 s. Sodium-desalted samples were vacuum dried in a SpeedVac Concentrator (Thermo Fisher Scientific). Samples were then dried three times with the consecutive additions of 200 μl of methanol to remove residual borate.

The desalted samples were further purified by homemade porous graphitized carbon (PGC) stage tips. Columns were created with 10 μl of PGC material from Extract Clean Carbograph cartridges in methanol, deposited onto a ZipTip C18 tip. The columns were conditioned by initially washing with elution buffer [80% (v/v) acetonitrile (ACN) and 0.1% (v/v) trifluoroacetic acid (TFA)] and subsequently equilibrated in loading buffer [0.1% (v/v) TFA]. Dried sample was dissolved in 50 μl of loading buffer and passed through the prepared columns, with flowthrough reloaded into the columns. Enriched glycans were eluted in 50 μl of elution buffer and vacuum dried. Dried, purified glycans were stored at −20°C until analysis using MS analysis.

Purified glycans were resuspended in 30 μl of water before MS analysis. Sample handling and injections were performed using Ultimate 3000 ultrahigh-performance liquid chromatography (Thermo Fisher Scientific). Samples were injected in loading buffer (10 mM NH_4_HCO_3_) through a PGC precolumn (3 μm, Hypercarb, 320 μm inside diameter × 100 mm) at a flow rate of 1 μl/min and subsequently through a PGC analytical column (3 μm, Hypercarb, 75 μm inside diameter × 100 mm) at a flow rate of 300 nl/min. Chromatographic separation was achieved using a 95-min gradient for *N*-glycans [0 to 70% (v/v) ACN]. The eluting glycans were detected using an amaZon electron transfer dissociation speed ion trap (Bruker) in the negative ion mode using a captive spray source. A mass/charge ratio range of 500 to 1800 was fixed for data-dependent precursor scanning. Both MS and tandem MS (MS/MS) data were recorded in the instrument’s ultrascan mode with an ion charge control target of 20,000 and accumulation time of 200 ms. Capillary exit voltage was set to 140 V, dry gas temperature at 77°C, and flow rate of 300 liters/min. High-voltage (HV) capillary was set to 1300 V, and HV end plate was offset to 500 V. Collision-induced dissociation fragmentation was performed on the five most intense precursors of each MS scan.

Data analyses were carried out in Compass Data Analysis 4.2 (Bruker Corporation) for manual glycan structural assignment and confirmation and in Byologic Software (Protein Metrics) for quantitation. Quantitation was performed for each isomer identified by MS/MS spectra and retention time by calculating the area under the curve.

### Statistical analyses

Raw, individual-level data are presented in data file S1. Statistical analyses were performed using Prism 9.0 (GraphPad). Comparisons between two groups were performed using paired or unpaired two-tailed Student’s *t* test. Differences among more than two groups were assessed using one- or two-way nonparametric ANOVA, followed by post hoc corrections for multiple comparisons (Tukey’s, Bonferroni’s, or Šidák’s). Survival data were analyzed using the log-rank (Mantel-Cox) or the Gehan-Wilcoxon test with post hoc Bonferroni’s correction for multiple comparisons, and the multivariate analysis of gene set 1 expression on patient survival was performed by Cox proportional hazard analysis. A *P* value <0.05 was considered statistically significant (*P ≤ 0.05, **P ≤ 0.01, ***P ≤ 0.001, and ****P ≤ 0.0001). *n* indicates the number of biological replicates, all bars within the graphs represent mean values, and the error bars represent SEMs.

## Supplementary Material

MDAR Reproducibility Checklist

Data file S1

Figs. S1 to S9, Tables S1 to S3, Legend for data file S1

## Data Availability

All data associated with this study are present in the paper or the Supplementary Materials. The scRNA-seq data generated and analyzed in this manuscript can be accessed at the Gene Expression Omnibus under accession number GSE208133. Code used for the analysis of the scRNA-seq data, as well as the TCGA gene expression database, is available at https://doi.org/10.5281/ZENODO.7116858. Reagents will be made available to the scientific community by contacting the corresponding authors and completion of a material transfer agreement.
