# Supplementary material for: Targeting cancer glycosylation repolarizes tumor-associated macrophages allowing effective immune checkpoint blockade
Source: Sci Transl Med. Author manuscript; Available in PMC 2023 Jan 5. (PMC9812757; doi:10.1126/scitranslmed.abj1270)
Supplement: MDAR Reproducibility Checklist [file NIHMS1858573-supplement-MDAR_Reproducibility_Checklist.pdf]

Materials Design Analysis Reporting (MDAR) Checklist for Authors

The MDAR framework establishes a minimum set of requirements in transparent reporting applicable to studies in the life sciences (see Statement of Task: doi:10.31222/osf.io/9sm4x.). The MDAR checklist is a tool for authors, editors, and others seeking to adopt the MDAR framework for transparent reporting in manuscripts and other outputs. Please refer to the MDAR Elaboration Document for additional context for the MDAR framework.

For all that apply, please note where in the manuscript the required information is provided.

Materials:

Newly created materials	indicate where provided: page no/section/legend)	n/a
The manuscript includes a dedicated "materials availability statement" providing transparent disclosure about availability of newly created materials including details on how materials can be accessed and describing any restrictions on access.	A data and material availability statement has been included.	

Antibodies	indicate where provided: page no/section/legend)	n/a
For commercial reagents, provide supplier name, catalogue number and RRID, if available.	We have added a list of all antibodies used in this study in the supplementary data file.	

DNA and RNA sequences	indicate where provided: page no/section/legend)	n/a
**Short novel DNA or RNA including primers, probes:** Sequences should be included or deposited in a public repository.	The DNA sequences used are included in the materials and methods section.	

Cell materials	indicate where provided: page no/section/legend	n/a
**Cell lines:** Provide species information, strain. Provide accession number in repository **OR** supplier name, catalog number, clone number, **OR** RRID.	All origins of cells are described in the material and methods section, *Cells and cell culture*.	
**Primary cultures:** Provide species, strain, sex of origin, genetic modification status.	All procedures have been described in the materials and methods section, *Mice*.	

Experimental animals	indicate where provided: page no/section/legend)	n/a
**Laboratory animals or Model organisms:** Provide species, strain, sex, age, genetic modification status. Provide accession number in repository **OR** supplier name, catalog number, clone number, **OR** RRID.	All origins of mouse lines have been described in the materials and methods section, *Mice*.	
**Animal observed in or captured from the field:** Provide species, sex, and age where possible.		na

Plants and microbes	indicate where provided: page no/section/legend)	n/a
**Plants:** provide species and strain, ecotype and cultivar where relevant, unique accession number if available, and source (including location for collected wild specimens).		na
**Microbes:** provide species and strain, unique accession number if available, and source.		na

Human research participants	indicate where provided: page no/section/legend) or state if these demographics were not collected	n/a
If collected and within the bounds of privacy constraints report on age, sex and gender or ethnicity for all study participants.		na

Design:

Study protocol	indicate where provided: page no/section/legend)		n/a
If study protocol has been pre-registered, provide DOI. For clinical trials, provide the trial registration number **OR** cite DOI.			n a

Laboratory protocol	indicate where provided: page no/section/legend)		n/a
Provide DOI **OR** other citation details if detailed step-by-step protocols are available.	All references for the used protocols have been provided in the materials and methods section.		

Experimental study design (statistics details)			
**For in vivo studies:** State whether and how the following have been done	**indicate where provided: page no/section/legend. If it could have been done, but was not, write not done**		**n/a**
Sample size determination	In order to achieve statistically significant results in regard to tumor growth, we aimed for a minimum group size of 5 mice for most of the presented experiments. Differences in tumor growth were measured by using a 2-way ANOVA with correction for multiple measurements.		
Randomisation	In order to avoid selection bias, transgenic mice were randomized to treatment arms before the injections were performed. In addition, mice were grouped based on tumor size at the time point of treatment.		
Blinding	Treatment teams in our laboratory always consist of 2 different researchers.		
Inclusion/exclusion criteria	Only healthy animals were included. Mice with no palpable tumor growth in tumor models were excluded from the experiment.		

Sample definition and in-laboratory replication	indicate where provided: page no/section/legend		n/a
State number of times the experiment was replicated in laboratory.	All statistical information is provided in the legend of each figure.		
Define whether data describe technical or biological replicates.	Provided numbers of replicates refer to biological replicates.		

Ethics	indicate where provided: page no/section/legend		n/a
**Studies involving human participants:** State details of authority granting ethics approval (IRB or equivalent committee(s), provide reference number for approval.	The details are mentioned in the materials and methods section, *Tumor digests*.		
**Studies involving experimental animals:** State details of authority granting ethics approval (IRB or equivalent committee(s), provide reference number for approval.	The details are given in the materials and methods section, *Mice*.		
**Studies involving specimen and field samples:** State if relevant permits obtained, provide details of authority approving study; if none were required, explain why.			n a

Dual Use Research of Concern (DURC)	indicate where provided: page no/section/legend		n/a
If study is subject to dual use research of concern regulations, state the authority granting approval and reference number for the regulatory approval.			n a

Analysis:

Attrition	indicate where provided: page no/section/legend	n/a
Describe whether exclusion criteria were preestablished. Report if sample or data points were omitted from analysis. If yes report if this was due to attrition or intentional exclusion and provide justification.	In mouse tumor experiments, mice carrying ulcerating tumors were excluded from further analyses. No other results were excluded from the analyses.	

Statistics	indicate where provided: page no/section/legend	n/a
Describe statistical tests used and justify choice of tests.	Statistical tests are described in the legend of each presented figure.	

Data availability	indicate where provided: page no/section/legend	n/a
For newly created and reused datasets, the manuscript includes a data availability statement that provides details for access or notes restrictions on access.	A data and material availability section has been included.	
If newly created datasets are publicly available, provide accession number in repository **OR** DOI **OR** URL and licensing details where available.	The deposited sequencing data can be accessed at GSE208133.	
If reused data is publicly available provide accession number in repository **OR** DOI **OR** URL, **OR** citation.	TCGA data was used (https://www.cancer.gov/about-nci/organization/ccg/research/structural-genomics/tcga).	

Code availability	indicate where provided: page no/section/legend	n/a
For all newly generated custom computer code/software/mathematical algorithm or re-used code essential for replicating the main findings of the study, the manuscript includes a data availability statement that provides details for access or notes restrictions.	A data and material availability section has been included.	
If newly generated code is publicly available, provide accession number in repository, **OR** DOI **OR** URL and licensing details where available. State any restrictions on code availability or accessibility.	All newly generated code has been deposited and can be found at https://doi.org/10.5281/ZENODO.7116858	
If reused code is publicly available provide accession number in repository **OR** DOI **OR** URL, **OR** citation.		na

Reporting

MDAR framework recommends adoption of discipline-specific guidelines, established and endorsed through community initiatives. Journals have their own policy about requiring specific guidelines and recommendations to complement MDAR.

Adherence to community standards	indicate where provided: page no/section/legend	n/a
State if relevant guidelines (e.g., ICMJE, MIBBI, ARRIVE) have been followed, and whether a checklist (e.g., CONSORT, PRISMA, ARRIVE) is provided with the manuscript.	No additional checklist is provided within the manuscript.	
