# Supplementary material for: Targeting cancer glycosylation repolarizes tumor-associated macrophages allowing effective immune checkpoint blockade
Source: Sci Transl Med. Author manuscript; Available in PMC 2023 Jan 5. (PMC9812757; doi:10.1126/scitranslmed.abj1270)
Supplement: Figs. S1 to S9, Tables S1 to S3, Legend for data file S1 [file NIHMS1858573-supplement-Figs__S1_to_S9__Tables_S1_to_S3__Legend_for_data_file_S1.pdf]

Science Translational Medicine
AAAS

Supplementary Materials for

Targeting cancer glycosylation repolarizes tumor-associated macrophages allowing effective immune checkpoint blockade

Michal A. Stanczak *et al.*

Corresponding author: Michal A. Stanczak, mstancz1@jhmi.edu; Heinz Läubli, heinz.laeubli@unibas.ch

Sci. Transl. Med. **14**, eabj1270 (2022)
DOI: 10.1126/scitranslmed.abj1270

The PDF file includes:

Figs. S1 to S9
Tables S1 to S3
Legend for data file S1

Other Supplementary Material for this manuscript includes the following:

Data file S1
MDAR Reproducibility Checklist

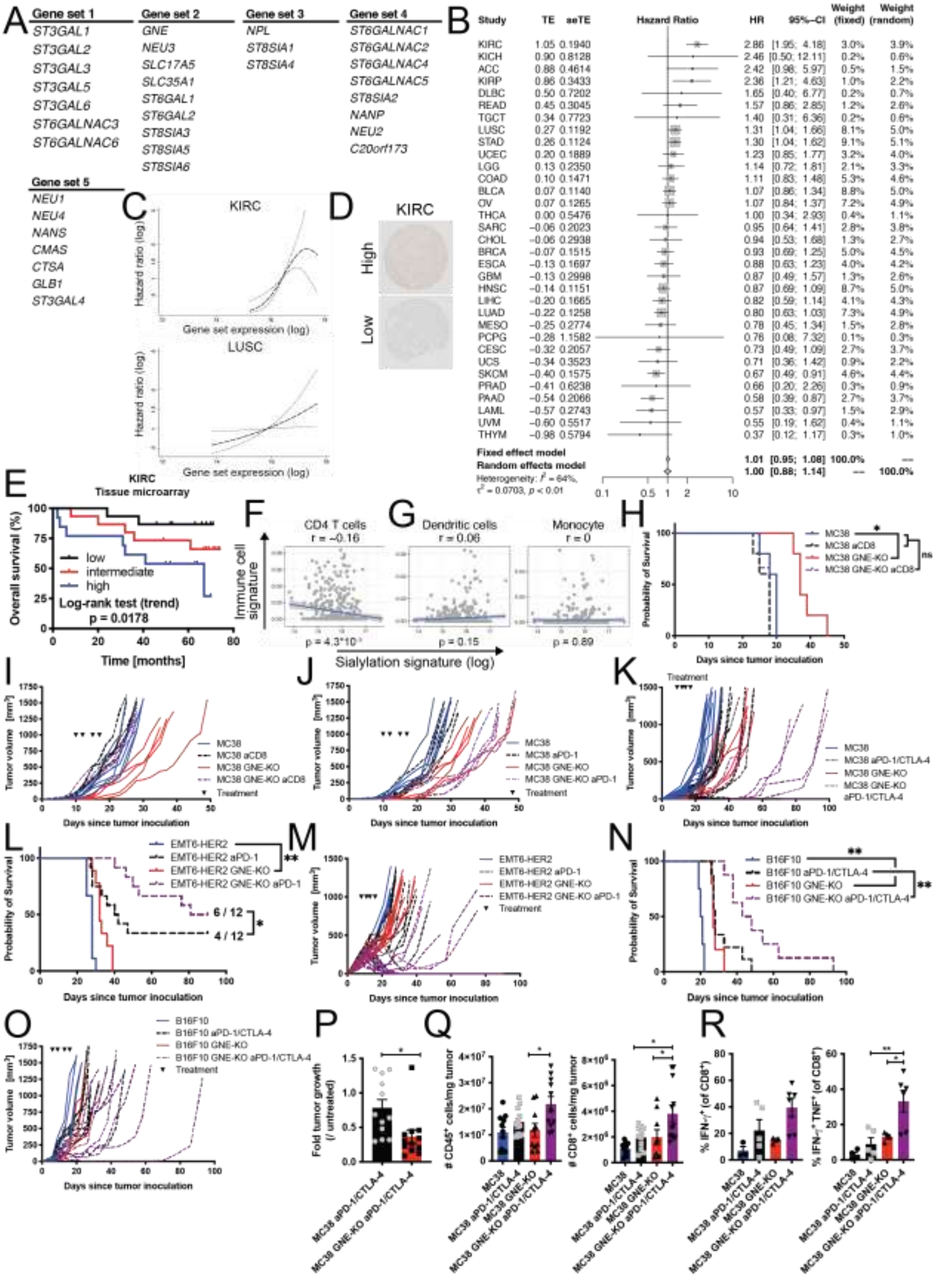
A
Gene set 1
ST3GAL1
ST3GAL2
ST3GAL3
ST3GAL5
ST3GAL6
ST6GALNAC3
ST6GALNAC6
Gene set 2
GNE
NEU3
SLC17A5
SLC35A1
ST6GAL1
ST6GAL2
ST8SIA3
ST8SIA5
ST8SIA6
Gene set 3
NPL
ST8SIA1
ST8SIA4
Gene set 4
ST6GALNAC1
ST6GALNAC2
ST6GALNAC4
ST6GALNAC5
ST8SIA2
NANP
NEU2
C20orf173
Gene set 5
NEU1
NEU4
NANS
CMAS
CTSA
GLB1
ST3GAL4
B
Study TE seTE Hazard Ratio HR 95%–CI Weight (fixed) Weight (random)
KIRC 1.05 0.1940 2.86 [1.95; 4.18] 3.0% 3.9%
KICH 0.90 0.8128 2.46 [0.50; 12.11] 0.2% 0.6%
ACC 0.88 0.4614 2.42 [0.98; 5.97] 0.5% 1.5%
KIRP 0.86 0.3433 2.36 [1.21; 4.63] 1.0% 2.2%
DLBC 0.50 0.7202 1.65 [0.40; 6.77] 0.2% 0.7%
READ 0.45 0.3045 1.57 [0.86; 2.85] 1.2% 2.6%
TGCT 0.34 0.7723 1.40 [0.31; 6.36] 0.2% 0.6%
LUSC 0.27 0.1192 1.31 [1.04; 1.66] 8.1% 5.0%
STAD 0.26 0.1124 1.30 [1.04; 1.62] 9.1% 5.1%
UCEC 0.20 0.1889 1.23 [0.85; 1.77] 3.2% 4.0%
LGG 0.13 0.2350 1.14 [0.72; 1.81] 2.1% 3.3%
COAD 0.10 0.1471 1.11 [0.83; 1.48] 5.3% 4.6%
BLCA 0.07 0.1140 1.07 [0.86; 1.34] 8.8% 5.0%
OV 0.07 0.1265 1.07 [0.84; 1.37] 7.2% 4.9%
THCA 0.00 0.5476 1.00 [0.34; 2.93] 0.4% 1.1%
SARC −0.06 0.2023 0.95 [0.64; 1.41] 2.8% 3.8%
CHOL −0.06 0.2938 0.94 [0.53; 1.68] 1.3% 2.7%
BRCA −0.07 0.1515 0.93 [0.69; 1.25] 5.0% 4.5%
ESCA −0.13 0.1697 0.88 [0.63; 1.23] 4.0% 4.2%
GBM −0.13 0.2998 0.87 [0.49; 1.57] 1.3% 2.6%
HNSC −0.14 0.1151 0.87 [0.69; 1.09] 8.7% 5.0%
LIHC −0.20 0.1665 0.82 [0.59; 1.14] 4.1% 4.3%
LUAD −0.22 0.1258 0.80 [0.63; 1.03] 7.3% 4.9%
MESO −0.25 0.2774 0.78 [0.45; 1.34] 1.5% 2.8%
PCPG −0.28 1.1582 0.76 [0.08; 7.32] 0.1% 0.3%
CESC −0.32 0.2057 0.73 [0.49; 1.09] 2.7% 3.7%
UCS −0.34 0.3523 0.71 [0.36; 1.42] 0.9% 2.2%
SKCM −0.40 0.1575 0.67 [0.49; 0.91] 4.6% 4.4%
PRAD −0.41 0.6238 0.66 [0.20; 2.26] 0.3% 0.9%
PAAD −0.54 0.2066 0.58 [0.39; 0.87] 2.7% 3.7%
LAML −0.57 0.2743 0.57 [0.33; 0.97] 1.5% 2.9%
UVM −0.60 0.5517 0.55 [0.19; 1.62] 0.4% 1.1%
THYM −0.98 0.5794 0.37 [0.12; 1.17] 0.3% 1.0%
Fixed effect model 1.01 [0.95; 1.08] 100.0% --
Random effects model 1.00 [0.88; 1.14] -- 100.0%
Heterogeneity: I^2 = 64%, τ^2 = 0.0703, p < 0.01
0.1 0.5 1 2 10
C
KIRC
Hazard ratio (log)
Gene set expression (log)
LUSC
Hazard ratio (log)
Gene set expression (log)
D
KIRC
High
Low
E
KIRC
Tissue microarray
Overall survival (%)
low
intermediate
high
Log-rank test (trend)
p = 0.0178
Time [months]
F
CD4 T cells
r = −0.16
p = 4.3*10⁻⁵
G
Dendritic cells
r = 0.06
p = 0.15
Monocyte
r = 0
p = 0.89
Immune cell signature
Sialylation signature (log)
H
MC38
MC38 aCD8
MC38 GNE-KO
MC38 GNE-KO aCD8
ns
Probability of Survival
Days since tumor inoculation
I
Tumor volume [mm³]
MC38
MC38 aCD8
MC38 GNE-KO
MC38 GNE-KO aCD8
Treatment
Days since tumor inoculation
J
Tumor volume [mm³]
MC38
MC38 aPD-1
MC38 GNE-KO
MC38 GNE-KO aPD-1
Treatment
Days since tumor inoculation
K
Treatment
Tumor volume [mm³]
MC38
MC38 aPD-1/CTLA-4
MC38 GNE-KO
MC38 GNE-KO aPD-1/CTLA-4
Days since tumor inoculation
L
EMT6-HER2
EMT6-HER2 aPD-1
EMT6-HER2 GNE-KO
EMT6-HER2 GNE-KO aPD-1
6 / 12
4 / 12
Probability of Survival
Days since tumor inoculation
M
Tumor volume [mm³]
EMT6-HER2
EMT6-HER2 aPD-1
EMT6-HER2 GNE-KO
EMT6-HER2 GNE-KO aPD-1
Treatment
Days since tumor inoculation
N
B16F10
B16F10 aPD-1/CTLA-4
B16F10 GNE-KO
B16F10 GNE-KO aPD-1/CTLA-4
Probability of Survival
Days since tumor inoculation
O
Tumor volume [mm³]
B16F10
B16F10 aPD-1/CTLA-4
B16F10 GNE-KO
B16F10 GNE-KO aPD-1/CTLA-4
Treatment
Days since tumor inoculation
P
Fold tumor growth (/ untreated)
MC38 aPD-1/CTLA-4
MC38 GNE-KO aPD-1/CTLA-4
Q
CD45⁺ cells/mg tumor
CD8⁺ cells/mg tumor
MC38
MC38 aPD-1/CTLA-4
MC38 GNE-KO
MC38 GNE-KO aPD-1/CTLA-4
R
% IFN-γ⁺ (of CD8⁺)
% IFN-γ⁺ TNF⁺ (of CD8⁺)
MC38
MC38 aPD-1/CTLA-4
MC38 GNE-KO
MC38 GNE-KO aPD-1/CTLA-4

Figure S1. Tumor sialylation is associated with immune suppression and reduced survival in patients with cancer.

(**A**) Gene sets 1 to 5 generated from the clustering of genes involved in sialic acid biosynthesis with immune genes using all solid cancers in The Cancer Genome Atlas (TCGA) database. (**B**) Forest tree plot showing hazard ratios of increasing gene set 1 expression for all solid cancer types in the TCGA. TE, treatment effect, log hazard ratio; seTE, standard error of treatment effect; HR, hazard ratio; CI, confidence interval. (**C**) Plot of continuous gene set 1 expression and the corresponding HRs in clear cell renal cell carcinoma (KIRC) and squamous cell carcinoma of the lung (LUSC). (**D**) Representative KIRC tissue cores of tumors with high and low intensities of Siglec-9 Fc staining, from a tissue microarray of 75 patients with KIRC**.** (**E**) Kaplan–Meier survival curve of patients with KIRC, divided into terciles based on the intensity of Siglec-9 Fc staining. (**F**) Correlation of gene set 1 expression with a gene expression signature of conventional $CD4^+$ T cells, for all patients with LUSC in the TCGA database. (**G**) Correlation of gene set 1 expression with a gene expression signature of dendritic cells (DCs) and monocytes, for all patients with LUSC in the TCGA database. (**H**) Impact of $CD8^+$ T cell depletion (aCD8) on the survival of mice carrying MC38 wildtype (WT) and UDP-GlcNAc 2-epimerase knockout (GNE-KO) tumors. (**I**) Impact of $CD8^+$ T cell depletion on the growth of individual MC38 WT and GNE-KO tumors. (**J**) Effect of anti-programmed cell death protein 1 (aPD-1) immune checkpoint blockade (ICB) on the growth of individual MC38 WT and GNE-KO tumors (H to J, n=5 to 6 mice per group). (**K**) Effect of anti-PD-1 and anti-cytotoxic T-lymphocyte-associated protein 4 (CTLA-4) ICB on the growth of individual MC38 WT and GNE-KO tumors (n=9 to 15 mice per group). (**L**) Effect of PD-1 blockade on the survival of mice bearing intramammary WT or GNE-KO EMT6-HER2 tumors. (**M**) Effect of an anti-PD-1 ICB on the growth of individual EMT6-HER2 WT and GNE-KO tumors (L and M, n=9 to 12 mice per group). (**N**) Effect of combined PD-1 and CTLA-4 blockade on the survival of mice bearing subcutaneous WT or GNE-KO B16F10 tumors (n=4 to 8 mice per group). (**O**) Effect of anti-PD-1 and anti-CTLA-4 ICB on the growth of individual B16F10 WT and GNE-KO tumors (N and O, n=4 to 8 mice per group). (**P**) Relative tumor growth over treatment period, compared to untreated. (**Q**) Absolute number of $CD45^+$ immune cells and $CD8^+$ T cells per mg of resected tumor. (**R**) Frequency of interferon (IFN)-γ^+ and IFN-γ^+ tumor necrosis factor $(TNF)^+$ $CD8^+$ T cells after ex vivo phorbol 12-myristate 13-acetate (PMA)/ionomycin restimulation (P to R, n=9 to 12 mice per group). Error bars represent the mean ± standard error of the mean (s.e.m.). Statistical analyses were performed using the log-rank (Mantel–Cox) test for the TCGA survival data (E) or the Gehan-Wilcoxon test for the mouse survival data (H, L, and

N), followed by Bonferroni's correction for multiple comparisons. An unpaired two-tailed Student's *t*-test was used in (P) and a one-way analysis of variance (ANOVA) followed by a post hoc Šidák's correction for multiple comparisons were used in (Q and R). ns, not significant; * $P \leq 0.05$, ** $P \leq 0.01$.

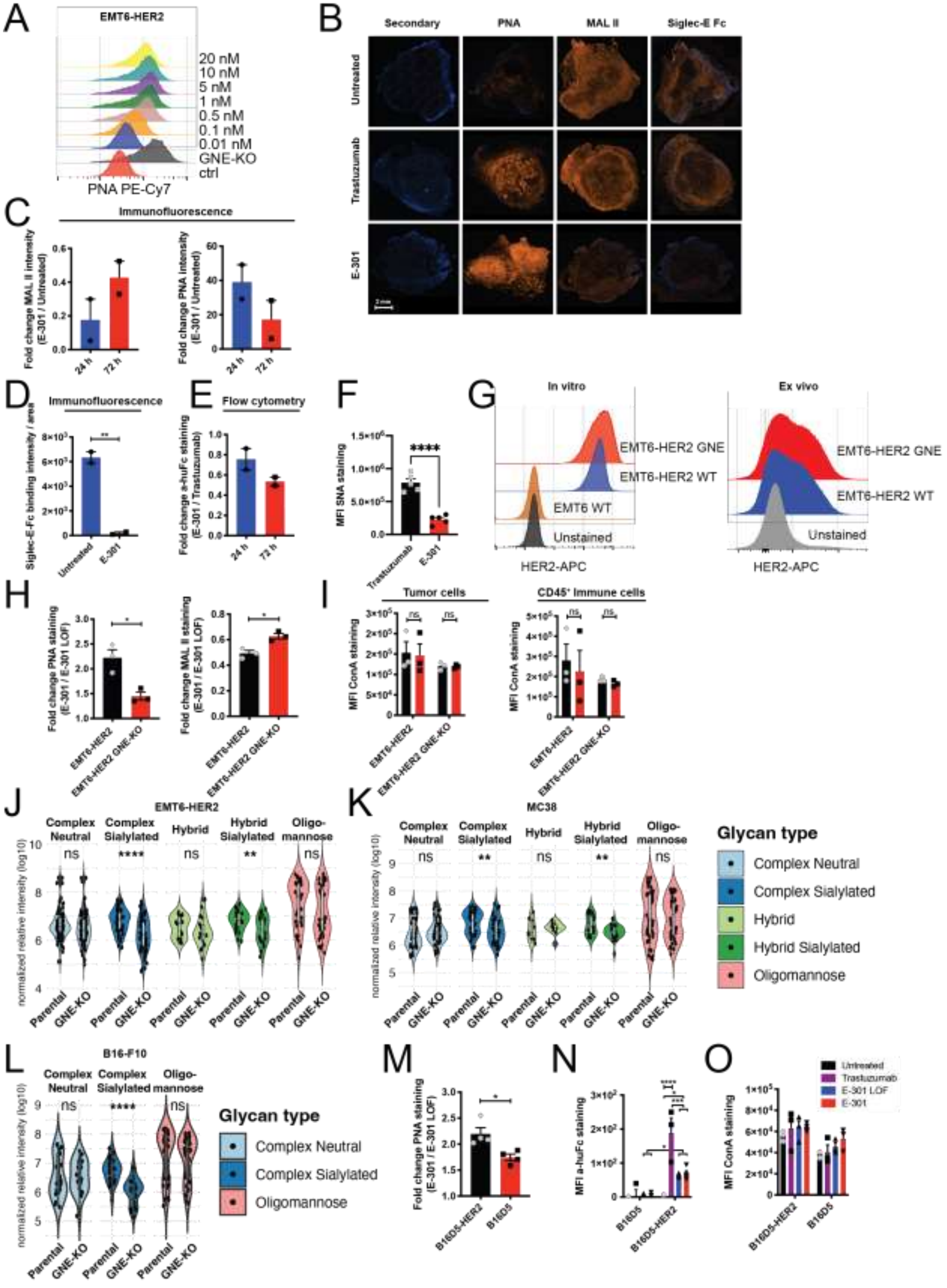
A
EMT6-HER2
20 nM
10 nM
5 nM
1 nM
0.5 nM
0.1 nM
0.01 nM
GNE-KO
ctrl
PNA PE-Cy7
B
Secondary
PNA
MAL II
Siglec-E Fc
Untreated
Trastuzumab
E-301
C
Immunofluorescence
Fold change MAL II intensity (E-301 / Untreated)
Fold change PNA intensity (E-301 / Untreated)
24 h
72 h
D
Immunofluorescence
Siglec-E-Fc binding intensity / area
Untreated
E-301
E
Flow cytometry
Fold change a-huFc staining (E-301 / Trastuzumab)
F
MFI SNA staining
Trastuzumab
E-301
G
In vitro
Ex vivo
EMT6-HER2 GNE
EMT6-HER2 WT
EMT6 WT
Unstained
HER2-APC
H
Fold change PNA staining (E-301 / E-301 LOF)
Fold change MAL II staining (E-301 / E-301 LOF)
EMT6-HER2
EMT6-HER2 GNE-KO
I
Tumor cells
CD45⁺ Immune cells
MFI ConA staining
J
EMT6-HER2
K
MC38
Complex Neutral
Complex Sialylated
Hybrid
Hybrid Sialylated
Oligo-mannose
normalized relative intensity (log10)
Parental
GNE-KO
Glycan type
Oligomannose
L
B16-F10
M
Fold change PNA staining (E-301 / E-301 LOF)
B16D5-HER2
B16D5
N
MFI a-huFc staining
O
Untreated
Trastuzumab
E-301 LOF
E-301
MFI ConA staining

Figure S2. Tumor-targeted sialidase effectively desialylates the tumor microenvironment.

(**A**) Representative histograms of peanut agglutinin (PNA) staining after a 24 hour in vitro incubation of EMT6-HER2 cells with increasing concentrations of trastuzumab, E-301 loss-of-function (LOF) or E-301. EMT6-HER2 GNE-KO cells were used as a control for desialylation. (**B**) Representative lectin and Siglec-E Fc stained immunofluorescence images of untreated, trastuzumab- or E-301-treated EMT6-HER2 tumors at 72 hours post-treatment. (**C**) Fold changes in *Maackia amurensis* lectin II (MAL II) and PNA staining intensities relative to those of the untreated control. (**D**) Quantification of immunofluorescence staining of Siglec-E Fc (72 hours). The sum of the staining intensity was normalized to the respective DAPI-stained area. (**E**) Fold change in the mean fluorescence intensity MFI of anti-human Fc staining 24 hours and 72 hours after E-301 treatment relative to that after trastuzumab treatment. For panels (B to E), n=2 mice per group. (**F**) MFI of *Sambucus Nigra* lectin (SNA) staining of EMT6-HER2 tumors 72 hours after treatment with trastuzumab or E-301 (n=5). (**G**) anti-HER2 staining of WT and GNE-KO EMT6-HER2 tumor cells, after in vitro culture and ex vivo isolated from established tumors. (**H**) Fold changes in PNA and MAL II staining intensities after E-301 treatment relative to E-301 LOF treated control tumors, in WT and GNE-KO EMT6-HER2 tumors. (**I**) MFI of Concavalin A (ConA) staining after E-301- or E-301 LOF-treatment of wildtype and GNE-KO EMT6-HER2 tumors, showing tumor cells and tumor-infiltrating $CD45^+$ immune cells. (**J**) liquid chromatography-mass spectrometry (LC-MS)-based N-glycan analysis of WT (parental) and GNE-KO EMT6-HER2 cells. (**K**) LC-MS-based N-glycan analysis of WT and GNE-KO MC38 cells. (**L**) LC-MS-based N-glycan analysis of WT and GNE-KO B16F10 cells, n=3 samples per group. (**M**) Fold change in the geometric MFI of PNA staining of B16D5 and B16D5-HER2 tumors after E-301 treatment relative to E-301 LOF treatment (n=4). (**N**) MFI of anti-human Fc (a-huFc) staining of B16D5 and B16D5-HER2 tumors after trastuzumab, E-301 LOF, or E-301 treatment. (**O**) MFI ConA staining of B16D5 and B16D5-HER2 tumors after trastuzumab, E-301 LOF, or E-301 treatment. For panels (N and O), n=3 to 4 replicates per group. Error bars represent the mean ± s.e.m. Statistical analyses were performed using unpaired two-tailed Student's *t*-tests in (C to H) and (M), or two-way ANOVAs followed by post hoc Šidák's corrections for multiple comparisons in (I and N). ns, not significant; * $P \leq 0.05$, ** $P \leq 0.01$, *** $P \leq 0.001$, **** $P \leq 0.0001$.

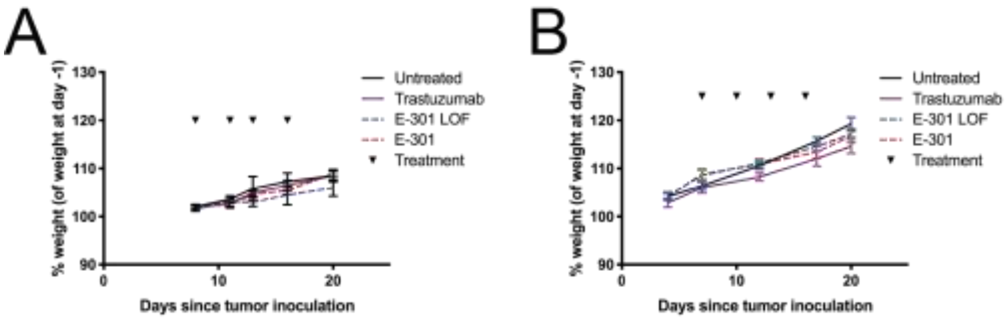

Figure S3. Tumor-targeted sialidase inhibits tumor growth by activating the adaptive immune system.

(**A**) Body weights of mice bearing intramammary EMT6-HER2 tumors after treatment with trastuzumab, E-301 LOF, or E-301 relative to weight before first treatment. (**B**) Body weights of mice bearing subcutaneous B16D5-HER2 tumors after treatment with trastuzumab, E-301 LOF, or E-301 relative to before treatment (n=6 to 8 mice per group). Arrowheads indicate treatment timing. Error bars represent the mean ± s.e.m.

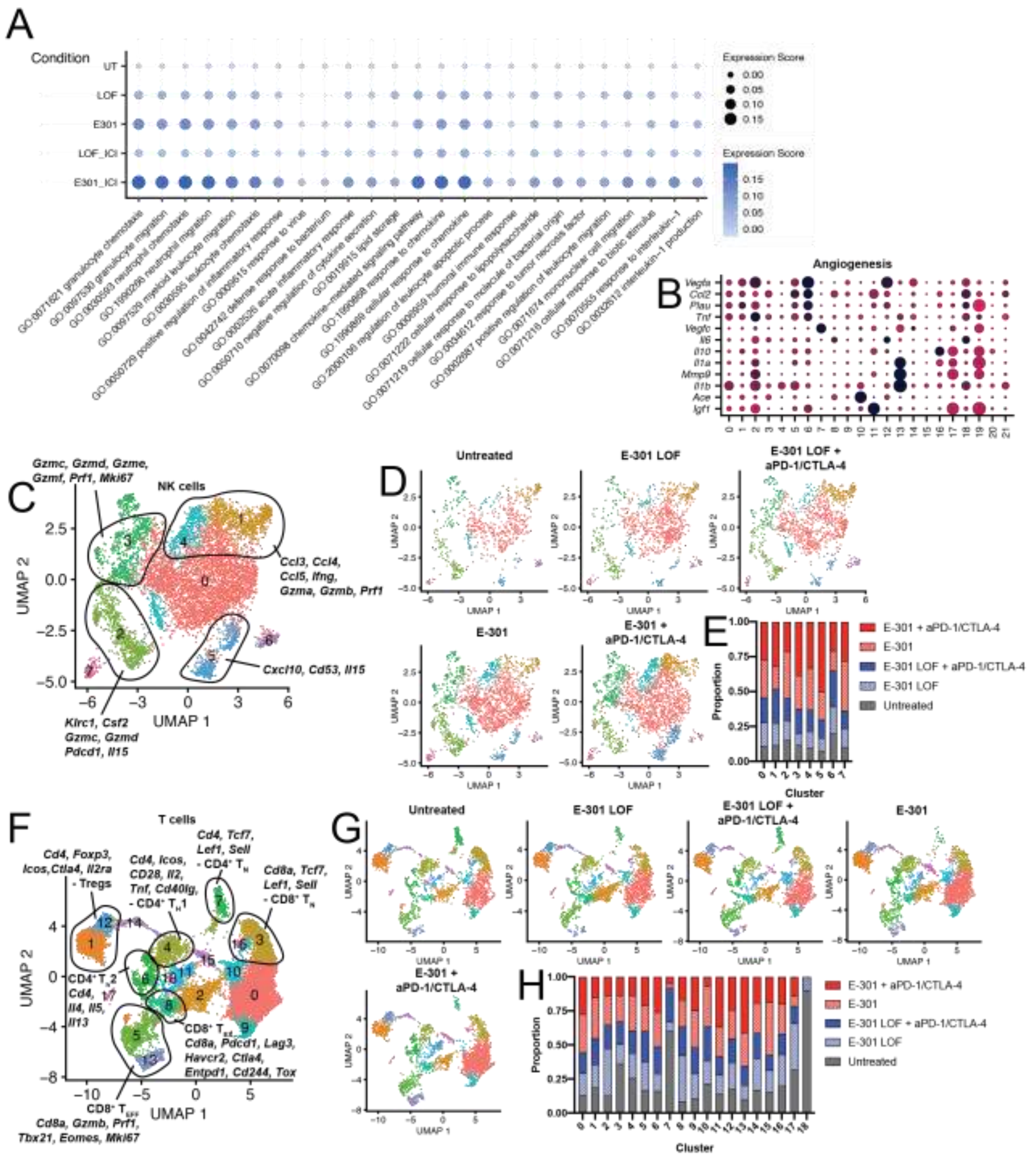

Figure S4. Therapeutic desialylation repolarizes tumor-associated macrophages (TAMs).

(**A**) Pathway enrichment analysis performed on all macrophages between treatment conditions. UT, untreated; LOF, E-301 LOF; LOF_ICI, E-301 loss of function plus immune checkpoint inhibition (ICI) with aPD-1/CTLA-4; E301_ICI, E-301 plus ICI with aPD-1/CTLA-4. (**B**) Dot plot representation of

differentially expressed genes between the macrophage clusters. Size reflects the percentage of each cluster expressing a given gene, average scaled expression is indicated on the color gradient. (**C**) Subclustering and uniform manifold approximation and projection (UMAP) projection of all natural killer (NK) cells. (**D**) UMAP projections of NK cells are shown separated by condition. (**E**) Contribution of each condition to each NK cell cluster. (**F**) Subclustering and UMAP projection of all T cells. (**G**) UMAP projections of T cells are shown separated by condition. (**H**) Contribution of each condition to each T cell cluster. For all panels, n=5 pooled mice per condition.

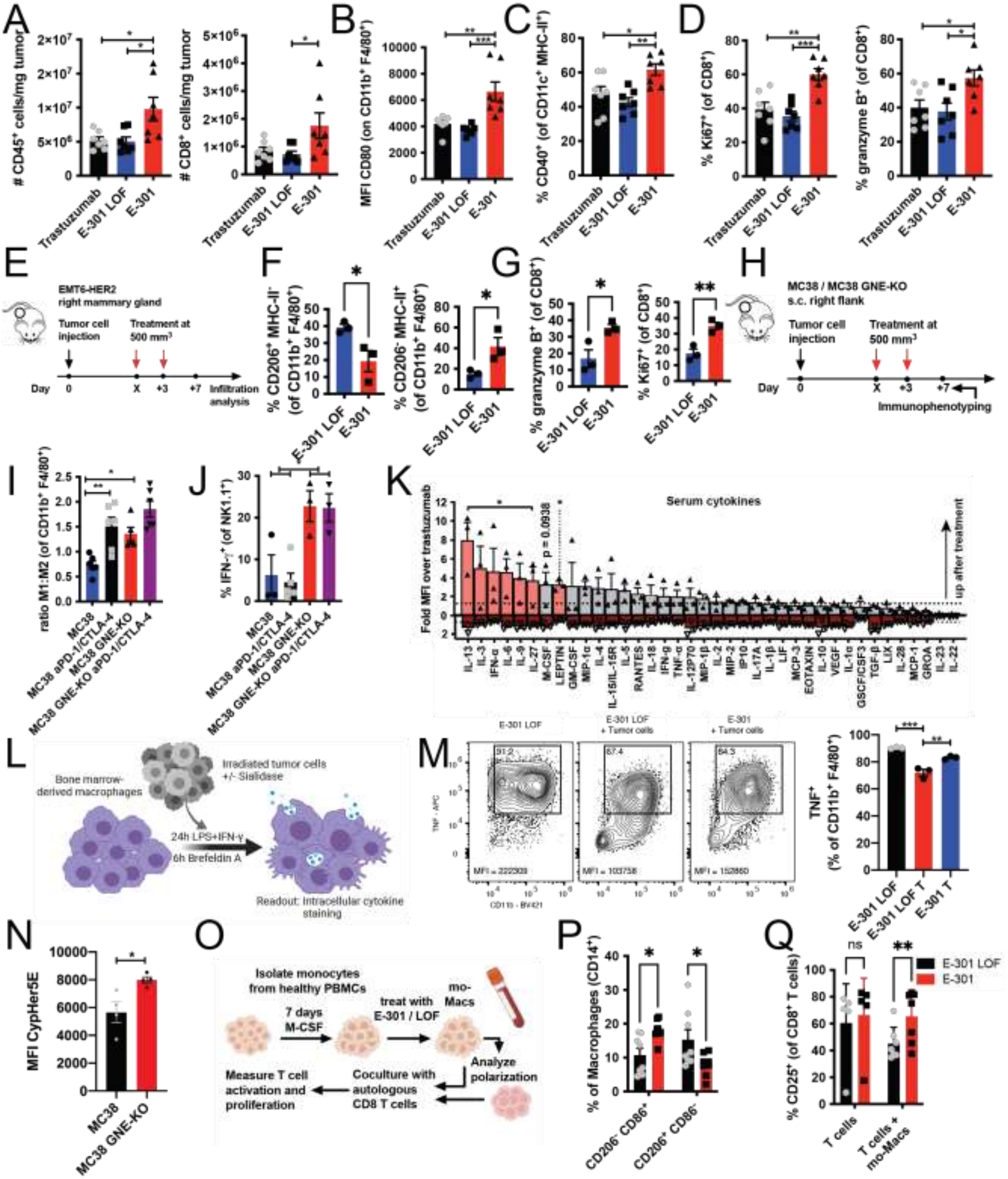

A
CD45+ cells/mg tumor
CD8+ cells/mg tumor
Trastuzumab
E-301 LOF
E-301
B
MFI CD80 (on CD11b+ F4/80+)
C
% CD40+ (of CD11c+ MHC-II+)
D
% Ki67+ (of CD8+)
% granzyme B+ (of CD8+)
E
EMT6-HER2
right mammary gland
Tumor cell injection
Treatment at 500 mm3
Day
Infiltration analysis
F
% CD206+ MHC-II- (of CD11b+ F4/80+)
% CD206- MHC-II+ (of CD11b+ F4/80+)
G
% granzyme B+ (of CD8+)
% Ki67+ (of CD8+)
H
MC38 / MC38 GNE-KO
s.c. right flank
Immunophenotyping
I
ratio M1:M2 (of CD11b+ F4/80+)
MC38
MC38 aPD-1/CTLA-4
MC38 GNE-KO
MC38 GNE-KO aPD-1/CTLA-4
J
% IFN-γ+ (of NK1.1+)
K
Serum cytokines
Fold MFI over trastuzumab
p = 0.0938
up after treatment
IL-13
IL-3
IFN-α
IL-6
IL-9
IL-27
M-CSF
LEPTIN
GM-CSF
MIP-1α
IL-4
IL-15/IL-15R
IL-5
RANTES
IL-18
IFN-g
TNF-α
IL-12P70
MIP-1β
IL-2
MIP-2
IP10
IL-17A
IL-1β
LIF
MCP-3
EOTAXIN
IL-10
VEGF
IL-1α
GSCF/CSF3
TGF-β
LIX
IL-28
MCP-1
GROA
IL-23
IL-22
L
Bone marrow-derived macrophages
Irradiated tumor cells +/- Sialidase
24h LPS+IFN-γ
6h Brefeldin A
Readout: Intracellular cytokine staining
M
E-301 LOF
E-301 LOF + Tumor cells
E-301 + Tumor cells
TNF - APC
CD11b - BV421
TNF+ (% of CD11b+ F4/80+)
E-301 LOF T
E-301 T
N
MFI CypHer5E
MC38
MC38 GNE-KO
O
Isolate monocytes from healthy PBMCs
7 days M-CSF
treat with E-301 / LOF
mo-Macs
Analyze polarization
Coculture with autologous CD8 T cells
Measure T cell activation and proliferation
P
% of Macrophages (CD14+)
CD206- CD86+
CD206+ CD86-
Q
% CD25+ (of CD8+ T cells)
ns
T cells
T cells + mo-Macs

Figure S5. Tumor desialylation repolarizes TAMs in murine and human tumors.

(**A**) Absolute number of total $CD45^+$ and $CD8^+$ T cells per mg of resected tumor. (**B)** MFI of CD80 expression on $CD11b^+F4/80^+$ TAMs. (**C**) Frequency of CD40 expression on DCs. (**D**) Frequency of granzyme B and Ki67 expression in $CD8^+$ T cells. For panels (A to D), n=7 murine tumor samples per group. (**E**) Experimental setup for immunophenotyping of changes in immune infiltrates after E-301 treatment: Mice bearing established (500 mm^3) subcutaneous EMT6-HER2 tumors were treated i.p. with two doses of 10 mg/kg trastuzumab, E-301 LOF, or E-301 and immune infiltrates were analyzed after 7 days by flow cytometry. (**F**) Frequencies of $CD206^+$ major histocompatibility complex $(MHC)\text{-}II^-$ (M2) and $CD206^-MHC\text{-}II^+$ (M1) cells among $CD11b^+F4/80^+$ TAMs. (**G**) Expression of granzyme B and Ki67 by $CD8^+$ T cells. For panels (F and G), n=3 mice per group. (**H**) Experimental design: Mice with established (approx. 500 mm^3) subcutaneous (s.c.) WT and GNE-KO MC38 tumors were treated i.p. with two doses of 10 mg/kg anti-PD-1 and anti-CTLA-4 antibodies. 7 days after the first treatment, tumors were resected and immunophenotyped. Same tumors as in Fig. 1K. (**I**) Ratio of M1 to M2 macrophages among $CD11b^+F4/80^+$ cells (n=5 to 6 mice per group). (**J**) Frequency of IFN-γ^+ NK cells after ex vivo PMA/ionomycin restimulation (n=3 to 5 samples per group). (**K**) Luminex analysis of cytokine concentrations in the serum of mice bearing subcutaneous B16D5-HER2 tumors treated with E-301, E-301 LOF, and trastuzumab at day 7 (n=3). Red bars of E-301 samples represent significant changes ($p < 0.05$). Dotted horizontal line indicates relative cytokine concentration in trastuzumab-treated control group. IL, interleukin; M-CSF, macrophage colony-stimulating factor; GM-CSF, granulocyte-macrophage colony-stimulating factor; MIP, macrophage inflammatory protein; RANTES, regulated upon activation, normal T cell expressed and secreted; IP-10, IFN-γ-induced protein 10 kDa; LIF, leukemia inhibitory factor; MCP3, monocyte-specific chemokine 3; VEGF, vascular endothelial growth factor; G-CSF, granulocyte colony-stimulating factor; LIX, lipopolysaccharide-induced CXC chemokine; MCP-1, monocyte chemoattractant protein-1; GROA, growth-regulated alpha protein. (**L**) Experimental setup for in vitro coculture of bone marrow-derived macrophages (BMDMs), irradiated B16D5-HER2 tumor cells, and sialidase. LPS, lipopolysachharide. (**M**) Representative dot plots of anti-TNF and anti-CD11b staining in cocultured BMDMs. Gates show percentages of TNF^+ cells, MFI indicates the mean fluorescence intensity of TNF of the TNF^+ population. The right panel shows the quantification of TNF^+ cells among $CD11b^+F4/80^+$ BMDMs (n=3). (**N**) Quantification of phagocytosis measured by CypHer5E intensity in $CD11b^+F4/80^+$ peritoneal macrophages after i.p. injection of CypHer5E labelled WT and GNE-KO MC38 tumor cells (n=4). (**O**) Experimental setup for in vitro coculture of primary human

monocyte-derived macrophages (mo-macs) from healthy peripheral blood mononuclear cells (PBMCs) and CD8 T cells. Monocytes were isolated from healthy PBMCs by adherence, differentiated into macrophages for 7 days in the presence of M-CSF, treated with E-301 LOF or E-301, and cocultured with autologous naïve CD8 T cells. (**P**) Flow cytometric analysis of TAM polarization in T cell cocultures after in vitro E-301 LOF or E-301 treatment. $CD206^{-}CD86^{+}$ M1 and $CD206^{+}CD86^{-}$ M2 macrophages are shown. (**Q**) Activation of CD8 T cells measured by CD25 expression, either after E-301 treatment of T cells alone or after coculture with autologous E-301 LOF- or E-301-treated mo-macs. For (P and Q), n=5 to 8 biological replicates. Error bars represent the mean ± s.e.m. Statistical analyses were performed using unpaired two-tailed Student's *t*-tests in (F, G, and N), as well as one- or two-way ANOVAs followed by post hoc Šidák's corrections for multiple comparisons in (A to D, I, J and M) or (P and Q), respectively. ns, not significant; * $P \leq 0.05$, ** $P \leq 0.01$, *** $P \leq 0.001$.

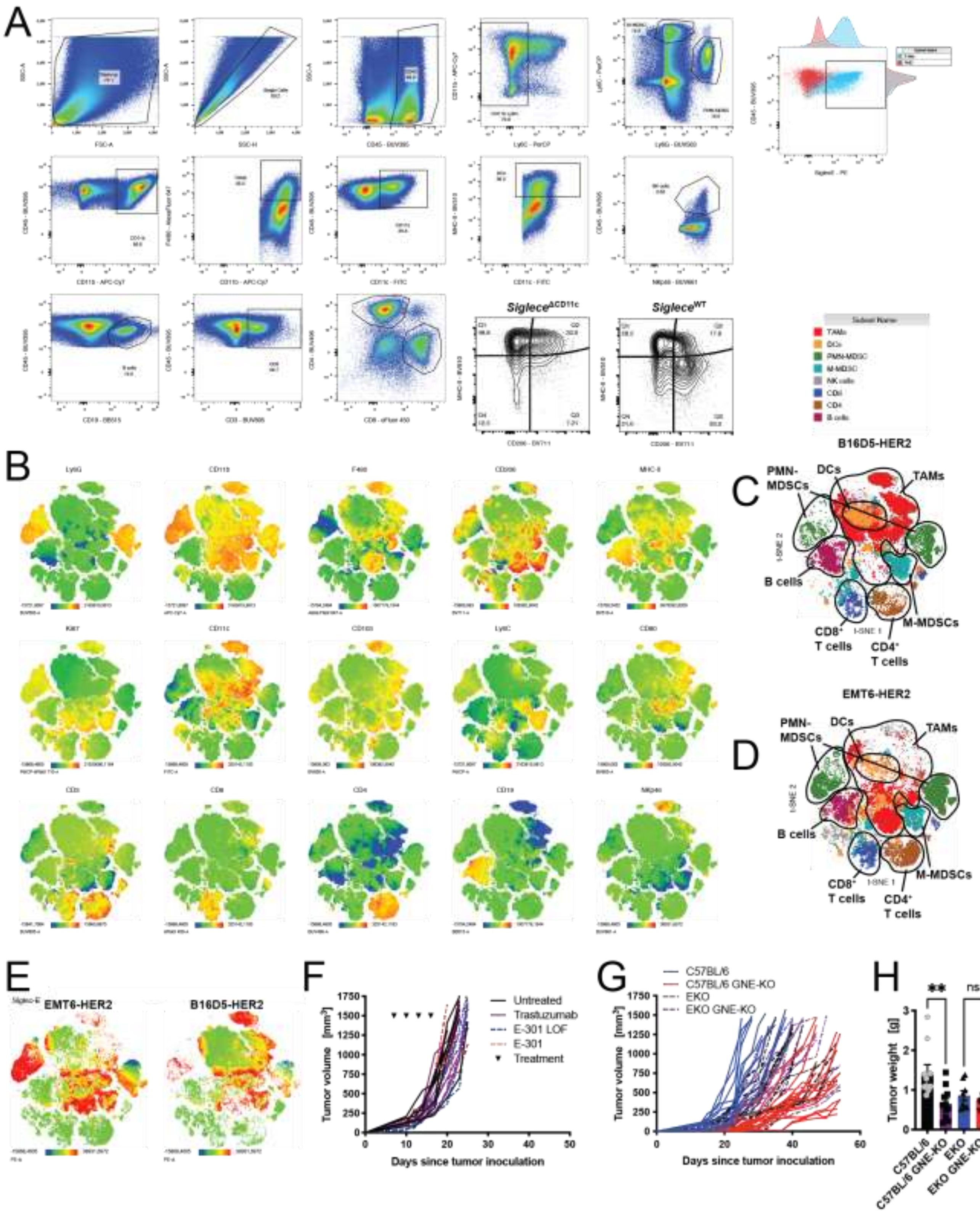

Figure S6. Efficacy of tumor-targeted sialidase is dependent on Siglec-E on TAMs.

(**A**) Exemplary gating strategy used for Fig. 6C. Representative Siglec-E staining and histogram shown on the right. Macrophage MHC-II and CD206 gating shown using representative M1 ($\textit{Siglece}^{\Delta CD11c}$) and M2 ($\textit{Siglece}^{\Delta WT}$) stainings. SSC-A, side scatter area; forward scatter area; SSC-H, side scatter height. FMO, fluorescence minus one control. (**B**) t-distributed stochastic neighbor embedding (t-SNE) projection of multicolor flow cytometric immunophenotyping of B16D5-HER2 and EMT6-HER2 tumors. Expression of individual markers is indicated as color gradients from blue (low) to red (high). (**C**) t-SNE projection of multicolor flow cytometric immunophenotyping of B16D5-HER2 tumors. Cell populations have been assigned based on maker expression. (**D**) t-SNE projection of multicolor flow cytometric immunophenotyping of EMT6-HER2 tumors. Cell populations have been assigned based on maker expression. (**E**) Staining intensity for Siglec-E is shown as a color gradient from blue (low) to red (high). Panels (A to E) show 5 concatenated samples per tumor type. (**F**) Growth of individual B16D5-HER2 tumors in Siglec-E knockout (EKO) mice after trastuzumab, E-301 LOF, or E-301 treatment (n=6 mice per group). (**G**) Growth of individual WT and GNE-KO MC38 tumors in C57BL/6 or EKO mice. (**H**) Tumor weights of endpoint MC38 WT and GNE-KO tumors from C57BL/6 and EKO mice. For panels (G and H), n=13 to 17 mice per group. Error bars represent the mean ± s.e.m. Statistical analyses were performed using a one-way ANOVA followed by post hoc Šidák's corrections for multiple comparisons in (H). ns, not significant. ** $P \leq 0.01$.

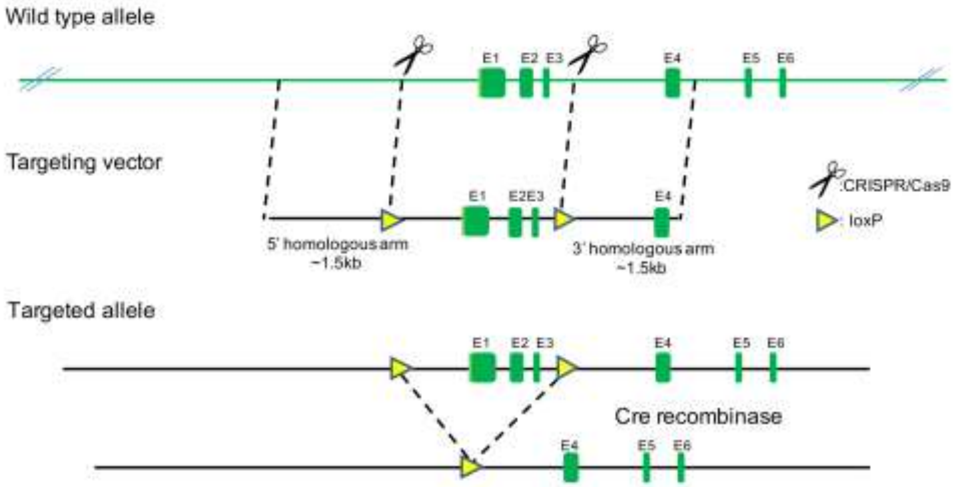

Figure S7. Generation of conditional *Siglece*$^{\mathbf{flox/flox}}$ mice.

Schematic workflow for the generation of conditional *Siglece*$^{flox/flox}$ mice. Exons 1 to 3 of *Siglece* were targeted by clustered regularly interspaced short palindromic repeats (CRISPR)/Cas9 and flanked with loxP sites.

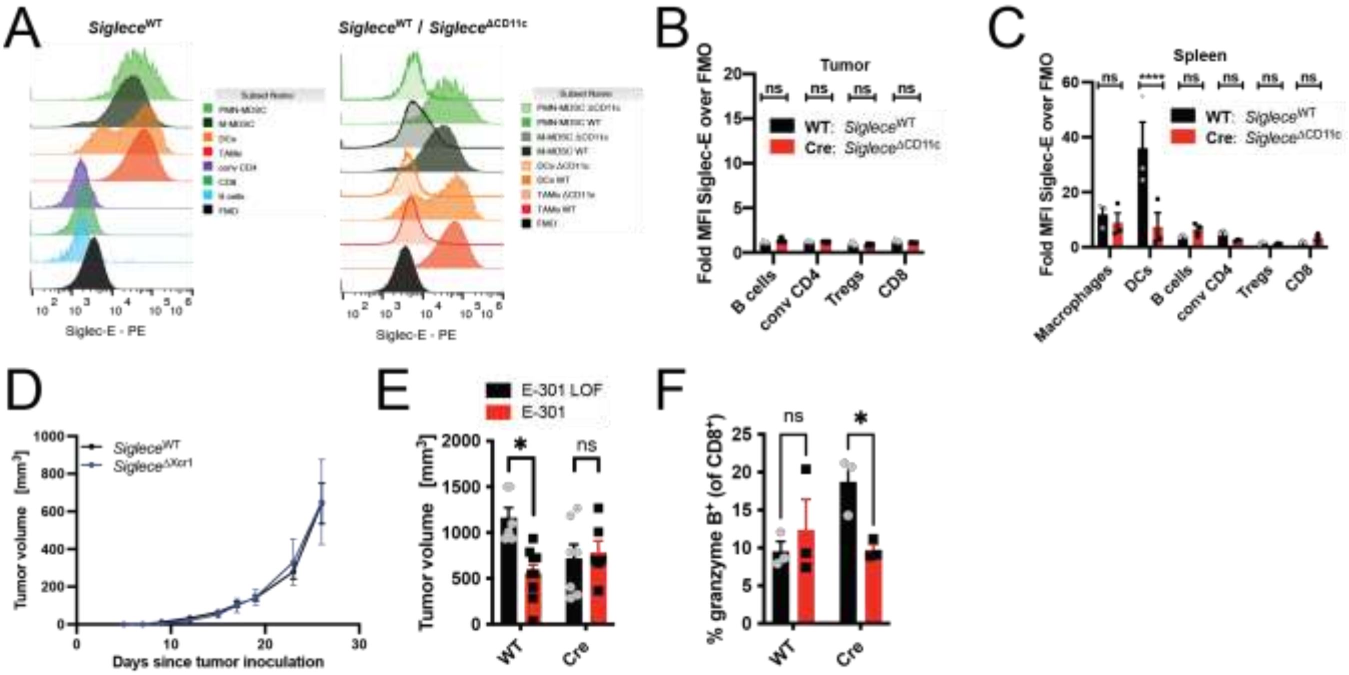

Figure S8. Efficacy of tumor-targeted sialidase is dependent on Siglec-E on TAMs.

(**A**) Representative histograms of anti-Siglec-E staining in different immune cell types from single-cell suspensions of MC38 tumors. Left: Baseline Siglec-E staining of immune cells from tumors grown in *Siglece*^{WT} mice. Right: Siglec-E staining of TAMs, DCs, M-MDSCs and PMN-MDSCs, grown in *Siglece*^{WT} (full histogram) or *Siglece*$^{\Delta\mathrm{CD11c}}$ (light histogram) mice. (**B**) Siglec-E expression by flow cytometry on different lymphoid tumor-infiltrating immune cells in *Siglece*$^{\Delta\mathrm{CD11c}}$ mice compared to littermate control mice. Siglec-E expression shown as fold change over FMO control staining (n=6 to 7 per group). (**C**) Siglec-E expression by flow cytometry on different splenic immune cells in *Siglece*$^{\Delta\mathrm{CD11c}}$ mice compared to littermate control mice. Siglec-E expression shown as fold change over FMO control staining (n=3 per group). (**D**) Tumor growth of subcutaneously injected MC38 cells in *Siglece*$^{\mathrm{flox/flox}}$ mice crossed to *Xcr1*^{cre} mice. *Siglece*$^{\Delta\mathrm{Xcr1}}$ are compared to *Siglece*^{WT} littermate controls (n=7 to 8 mice per group). (**E**) Tumor volumes of E-301 LOF- or E-301-treated *Siglece*$^{\Delta\mathrm{CD11c}}$ and *Siglece*^{WT} mice on day 29 after subcutaneous injection of B16D5-HER2 cells (n=7 to 9 mice per group). (**F**), CD8 T cell activation after coculture with TAMs from *Siglece*$^{\Delta\mathrm{CD11c}}$ or *Siglece*^{WT} mice and E-301 treatment (n=3 mice per group). Error bars represent the mean ± s.e.m. Statistical analyses were performed using two-way ANOVAs followed by post hoc Šidák's corrections for multiple comparisons. ns, not significant. * $P \leq 0.05$, **** $P \leq 0.0001$.

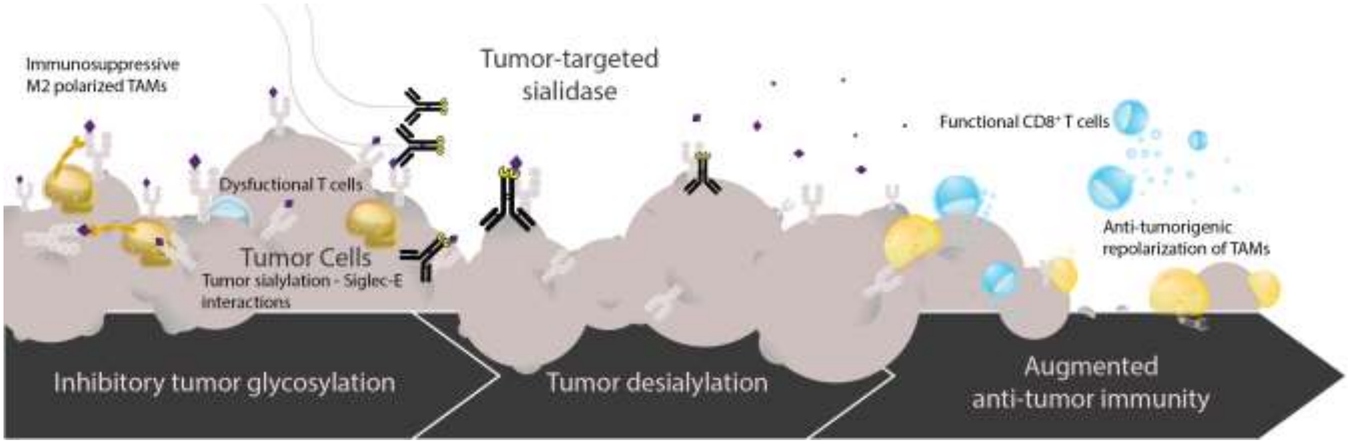

Figure S9. Graphical summary.

Table S1. Anti-mouse fluorophore-conjugates antibodies. BUV, brilliant ultraviolet; UV, ultraviolet; BV, brilliant violet; SB, super bright; BB, brilliant blue; FITC, fluorescein isothiocynate; PerCP, peridinin chlorophyll protein; PE, phycoerythrin; AF, alexa fluor; APC, allophycocyanin.

	Color	Dilution	Vendor	Cat. Number
**CD45**	BUV395	1:100	BD Biosciences	564279
**Live-dead**	Zombie UV	1:100	BioLegend	423107
**CD4**	BUV496	1:100	BD Biosciences	93937
**Ly-6G**	BUV563	1:200	BD Biosciences	612921
**NKp46**	BUV661	1:70	BD Biosciences	741678
**CD3**	BUV805	1:70	BD Biosciences	749276
**PD-L1**	BV421	1:150	BioLegend	124315
**LFA-1**	SB436	1:100	eBioscience	62011180
**CD8**	eFluor 450	1:100	eBioscience	480081
**MHCII**	BV510	1:300	BioLegend	107635
**CD80**	BV605	1:70	BioLegend	104729
**CD103**	BV650	1:70	BD Biosciences	748256
**CD206**	BV711	1:100	BioLegend	141727
**PD-1**	BV785	1:100	BioLegend	135225
**CD19**	BB515	1:100	BD Biosciences	564509
**CD11c**	FITC	1:100	BioLegend	117306
**Ly-6C**	PerCP	1:200	BioLegend	128028
**Tim-3**	BB700	1:100	BD Biosciences	747619
**Siglec E**	PE	1:50	BioLegend	677104
**CD25**	PE-Cy5.5	1:100	eBioscience	35025182
**F4/80**	AF647	1:100	BioLegend	123122
**CD11b**	APC-Cy7	1:100	BioLegend	101226
**Ki67**	AF532	1:200	eBioscience	58569882
**TCF-7**	AF700	1:100	R&D systems	FAB82224N
**GzmB**	PE-eFluor610	1:100	eBioscience	61889882
**FoxP3**	APC	1:100	eBioscience	17577382

Table S2. Anti-human fluorophore-conjugated antibodies. CTV, CellTrace Violet.

	Color	Dilution	Vendor	Cat. Number
**Live-dead**	Zombie UV	1:100	BioLegend	423107
**CD8**	BV805	1:100	BD Biosciences	749366
**CTV**	CTV	1:3000	Invitrogen	C34557
**CD25**	BV605	1:100	BioLegend	302632
**CD86**	BV711	1:100	BioLegend	305440
**MHCII**	FITC	1:200	BioLegend	307604
**CD4**	PerCP-Cy5.5	1:100	BioLegend	344608
**CD206**	PE	1:100	BioLegend	321106
**CCR7**	Pe-Cy7	1:100	BD Biosciences	557648
**CD14**	APC	1:200	BioLegend	367118
**CD163**	APC-Cy7	1:100	BioLegend	333662

Table S3. T cell activation antibodies

	Dilution	Vendor	Cat. Number
Anti mouse CD3	2.5 μg/ml	BioLegend	100202
Anti mouse CD28	5 μg/ml	BD Biosciences	553295
Anti-human CD3/CD28/CD2	25 μl/ml	Stemcell	10970

Data file S1. Raw, individual-level data for experiments where n<20.
